# Epidemiology of a Hybrid Swarm: Evidence of 11 Feline Infectious Agents Circulating in a Population of Sympatric European Wildcat Hybrids and Free-Living Domestic Cats, in Scotland

**DOI:** 10.1155/2023/6692514

**Published:** 2023-10-05

**Authors:** Beatriz S. G. Alves, Alice Bacon, Keri Langridge, Kostas Papasouliotis, Ian Handel, Neil E. Anderson, Anna L. Meredith

**Affiliations:** ^1^The Royal (Dick) School of Veterinary Studies (R(D)SVS) and The Roslin Institute, University of Edinburgh, Easter Bush Campus, Midlothian, Edinburgh EH25 9RG, UK; ^2^Faculdade de Ciências da Universidade do Porto, Rua do Campo Alegre, s/n, Porto 4169-007, Portugal; ^3^BIOPOLIS/CIBIO-Centro de Investigação em Biodiversidade e Recursos Genéticos, Universidade do Porto, Campus de Vairão, Rua Padre Armando Quintas no. 7, Vairão 4485-661, Portugal; ^4^Saving Wildcats, Royal Zoological Society of Scotland, Conservation Department, Highland Wildlife Park, Kincraig, UK; ^5^Scottish Wildcat Action, Great Glen House, Leachkin Road, Inverness IV3 8NW, UK; ^6^Langford Vets–Bristol Veterinary School, University of Bristol, Langford House Langford, Bristol BS40 5DU, UK

## Abstract

Hybridisation between wild and domestic species poses a serious challenge to conservation management and can, potentially, lead to extinction. Alongside it, disease transmission will inevitably occur. However, the link between these two phenomena has historically been neglected. In Scotland, the European wildcat is particularly threatened by hybridisation with the domestic cat, a process promoted by long-term habitat loss, human encroachment, and persecution. Between 2015 and 2019, free-living cats (*n* = 120) were captured in six conservation priority areas of northern Scotland. Samples were collected for infectious disease screening (feline immunodeficiency virus, feline leukaemia virus, feline calicivirus, feline herpesvirus, *Chlamydia felis*, *Mycoplasma felis*, *Bordetella bronchiseptica*, *Mycoplasma haemofelis*, Candidatus *Mycoplasma haemominutum*, Candidatus *Mycoplasma turicensis*, and *Tritrichomonas foetus*) and genetic analysis. Polymerase chain reaction and reverse transcriptionPCR were used to detect infectious DNA or RNA, respectively. The hybrid score (Q) for each individual cat was determined using a 35-SNP-marker test. Statistical analysis investigated the association between Q and probability of infection, accounting for spatial clustering. The results confirmed the presence of 11 infectious agents circulating in the free-living cat population of northern Scotland, which consists of a hybrid swarm between *F. silvestris* and *F. catus*. For eight of them (feline leukaemia virus, feline herpesvirus *C. felis*, *B. bronchiseptica*, *M. felis*, *M. haemofelis*, Ca. *M. haemominutum*, and *T. foetus*), there was no significant association between infection probability and Q, supporting our hypothesis that the hybrid swarm may be functioning as a single epidemiological unit. Considering the impact of infectious diseases on health, welfare, and population dynamics of domestic cats, their presence in the extremely fragile and hybridised population of *F. silvestris* in Scotland could be population limiting or, potentially, contribute to local extinction. Comprehensive disease surveillance, risk analysis, and domestic cat management will be essential for the European wildcat conservation, particularly where hybridisation is increasing and anthropogenic factors are prevalent.

## 1. Introduction

Wildlife conservation increasingly requires the understanding and management of the disease threats. Wild animals have coevolved with infectious agents, which are an inevitable part of a functional and balanced ecosystem. However, this equilibrium has been challenged by an expanding human population and the consequent anthropogenic impacts on the wildlife habitats [1]. Concurrently, hybridisation between domestic and wild species can pose severe threats to wildlife conservation. Human-induced hybridisation, frequently associated with species introductions and habitat degradation, may promote reproductive opportunities between taxa for which natural interbreeding would be highly unlikely [2]. In the United Kingdom alone, recent studies have demonstrated genetic evidence of introgression of wild mammals [3, 4] and birds [5] with their domestic counterparts. Matias et al. [2] analysed data from 13 European countries and found European wildcats (*Felis silvestris*) to, generally, have genetic integrity levels above the wildcat-hybrid threshold (ca. 83%; threshold = 80%). However, Mediterranean and Temperate Insular biomes (i.e., Scotland) revealed significantly lower levels, with 74% and 46% expected genetic integrity, respectively.

Since the 18^th^ century, wildcats in Scotland have experienced habitat loss, human encroachment, and persecution, resulting in very small and fragmented populations [6]. Consequently, wildcat individuals are more likely to encounter domestic cats (*Felis catus*) than members of their own species, particularly where domestic cat densities are high [7, 8]. As a result, hybridisation and potential disease transmission are more likely to occur between the two species [7, 9, 10].

Hybridisation is currently considered as primary threat for the wildcat in Scotland [8], with an estimated level of introgression close to 100%, leading to the assumption that no genetically distinct wildcats remain in the wild [3]. Instead, Senn et al. [3] describe the contemporary Scottish wild-living cat population as a “hybrid swarm,” consisting of a genetic continuum between *F. silvestris* and *F. catus*. In this study, it was concluded that hybrids have become so common that they mate with each other, producing more complex hybrids. Therefore, the population of wild-living cats in Scotland constitutes a swarm of genetically intermediate types, not displaying the more bimodal distribution of hybrid scores, typical of other systems where hybridisation is rare [3]. In this context, the question is raised of whether, in terms of disease transmission, this population may also act as a single epidemiological unit. Overall, disease transmission dynamics in hybridised populations are complex and can vary greatly depending on the species involved, the pathogen in question and the ecological context. Understanding these dynamics is important for both conservation efforts and managing potential disease risks within these populations. The hybridised population might act as a bridge for infectious disease transmission between the wild and domestic species, facilitating the spread of pathogens [11]. Where the two species are still evidently distinct and separate, differences are expected in terms of disease prevalence and distribution between them. However, we hypothesise that, in the case of a hybrid swarm, the interactions between the wild and domestic species that resulted in this genetic continuum, may also have allowed a more homogenous transmission of pathogens, thus leading to a potential single epidemiological unit. In a recent study by Smith et al. [11], the investigation of the epidemiology of *Trichomonas*, at an avian wild–feral–domestic interface where hybridisation occurs, suggested that individual infection status was not explained by the hybrid score (although this was assessed visually, not through genetic analysis).

Infectious disease research in *F. silvestris* has been conducted in several range countries, namely France [12–16], Scotland [17–19], Switzerland [13], Germany [13], Slovenia [20], Spain [21], Portugal [22], and Luxembourg [23]. These studies have demonstrated infection by and/or exposure to feline pathogens, which commonly cause significant clinical disease in the domestic cats, in wildcat populations across Europe. They mainly investigated viral infections that could be transmitted by the sympatric carnivores, particularly the domestic cat. However, most of these studies focus on putative wildcats (either genetically confirmed or phenotypically presumed), not including sympatric hybrids and domestic cats (namely, free-ranging pet cats, stray, and feral cats, who may represent different epidemiological impacts and implications). This approach considerably limits our understanding of these infectious agents, particularly since the domestic cat may act as their reservoir. As hybridisation escalates and contact between domestic cats, wildcats, and hybrids becomes more frequent, it is possible that, epidemiologically, they start acting as one single population.

We aimed to increase the understanding of feline infectious diseases in the free-living cat population of Scotland, to inform future European wildcat conservation strategies. We hypothesised that: (i) the hybrid swarm described by Senn et al. [3] may effectively constitute a single epidemiological unit, with no significant differences in infection probability across the hybrid scale; (ii) different risk factors known to affect the likelihood of infection in the domestic cats (as well as individual cat health and population dynamics), may also be involved in the epidemiology of wildcats and hybrids. To test these hypotheses, we modelled the association between hybrid score (hereafter Q) and probability of infection, accounting for spatial clustering. Subsequently, for infections found to have a statistically significant association with Q, we included in the model other risk factors (social system, age, sex, and body condition score (BCS)), in order to assess if their inclusion would influence the effect of Q. We further discuss the potential threat that feline infectious diseases pose to the conservation of *F. silvestris* and propose recommendations for the future research and standardised disease surveillance across the species' range.

## 2. Materials and Methods

### 2.1. Scottish Wildcat Action (SWA)

Launched in 2015, Scottish Wildcat Action (SWA) was the first national conservation project for *F. silvestris* in Scotland, aiming to halt the species decline within 5 years, by delivering *in situ* and *ex situ* management actions [24]. In order to assess the risks posed by feral domestic cats to wildcats, SWA conducted infectious disease screening of free-living cats (wildcats, feral domestic cats, and hybrids). This disease surveillance programme constitutes the basis of the present study.

### 2.2. Study Area

**Figure 1:**
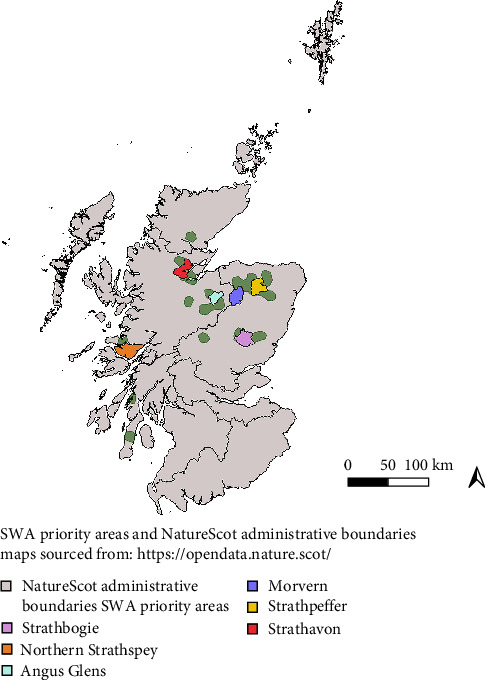
The six wildcat conservation priority areas initially identified by Scottish Wildcat Action, within NatureScot administrative boundaries. Green areas represent the current European wildcat presumptive distribution, according to the IUCN Red List.

The study area included the initial six conservation wildcat priority areas (hereafter ‘PAs') identified by SWA, after extensive camera-trap surveying across northern Scotland (Figure 1): “Morvern” (MV), “Strathpeffer” (SP), “Northern Strathspey” (SS), “Strathbogie” (SB), “Angus Glens” (AG), and “Strathavon” (SA) [24]. Field work at Strathavon was halted after 2 years, due to the absence of wildcats or high-scoring hybrids.

### 2.3. Study Design

A cross-sectional study was carried out to investigate 11 feline infectious agents–feline immunodeficiency virus (FIV), feline leukaemia virus (FeLV), feline calicivirus (FCV), feline herpesvirus (FHV), *Chlamydia felis*, *Mycoplasma felis*, *Bordetella bronchiseptica*, *Mycoplasma haemofelis*, Candidatus *Mycoplasma haemominutum*, Candidatus *Mycoplasma turicensis*, and *Tritrichomonas foetus*. Live free-living cats, including wildcats, feral domestic cats, and domestic-wildcat hybrids (from F1 progeny to backcrossed individuals with varying levels of wild and domestic ancestry), were captured, between 2015 and 2019, using cage-traps and complying with national licensing requirements and animal welfare standards [25]. Based on Kitchener et al. [26], the pelage of each cat was scored and cats were classified as either “domestic or low-scoring hybrids” (score of 7-16/21) or “wildcats or high-scoring hybrids” (scores of >17/21). Cats belonging to the first group were included in a trap-neuter-vaccinate-return (TNVR) scheme, including neutering, vaccination, and sample collection for genetics and disease screening [25]. In addition to sample collection, some of the cats that were scored as “wildcats or high-scoring hybrids” were micro-chipped and fitted with telemetry collars prior to release (from 2018 onwards; [27]).

Samples from each individual cat were submitted for infectious disease screening, according to Table 1. Polymerase chain reaction (PCR) allowed the detection of the infectious agents' DNA or RNA (in the case of reverse transcription PCR (RT–PCR) for FCV). Laboratory tests were conducted at Langford Vets Diagnostic Laboratories, University of Bristol. A blood sample was collected for genetic analysis, to determine the individual cats' hybrid score (Q). Q, allocated by the Bayesian population assignment programme STRUCTURE [39, 40], consists of a numeric variable (ranging from 0, domestic cat, to 1, wildcat), representing the estimated posterior probability of the cat being a wildcat. Each Q value has an associated 90% posterior credibility interval (CI), with LBQ = lower boundary of the 90% CI; and UBQ = upper boundary of the 90% CI. The analysis is based on a 35 nuclear single-nucleotide-polymorphism (SNP) marker test, designed to assess hybridisation between wildcat and domestic cat populations in Scotland [3]. The thresholds are defined as: LBQ ≥ 0.75 = wildcat; UBQ ≤ 0.25 = domestic cat; LBQ > 0.25, and UBQ < 0.75 = hybrids. Genetic analysis was conducted at the Royal Zoological Society of Scotland Wildgenes Laboratory.

Raw data (infectious agent screening results, Qs, and associated metadata) were entered and archived into the SWA Access database. Data cleaning was performed by SWA and only cats for which it was possible to gather comprehensive metadata (*n* = 120) were included in the analysis. From the original dataset, six independent variables/risk factors were extracted, to be used in the statistical analysis: one continuous, Q; and five categorical, SWA priority area (SWA PA), social system, age, sex, and BCS (Table 2). The infectious agents tested correspond to 11 binomial dependent variables. Results are presented as “Negative” or “Positive”. “NA” was applied in cases where it was not possible to obtain samples or the test result was unreliable. All “NA” cases were excluded from each step of the statistical analysis. Q is presented as a numeric value (0–1), as described previously.

### 2.4. GIS Mapping and Analysis

**Figure 2:**
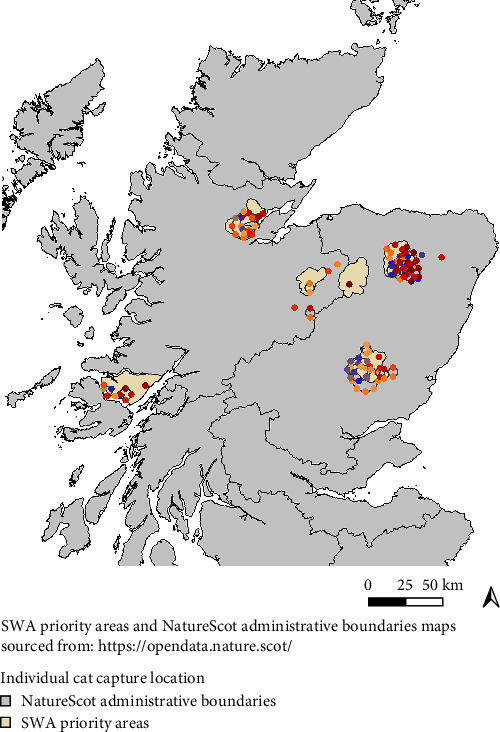
Map displaying the locations of all the individual cats (*n* = 120) included in the analysis. Each circle represents a single cat and the colour gradient reflects the Q score as a continuous variable (red–orange–blue gradient, from lower to higher Q score or from domestic to wildcat).

Geographical locations of individual cats were mapped using QGIS software, version 3.12.0 [42] (Figure 2). Overlapping points were jittered to facilitate ease of viewing. The shapefile layers were sourced from the Natural Spaces-Scottish Natural Heritage website [43].

### 2.5. Statistical Analysis

The statistical analysis was conducted using the statistical software R, version 3.6.2. [44], within RStudio, version 1.2.5033 [45]. Specific R packages will be referred to in the following sections and R script for models can be found in supplementary materials.

### 2.6. Descriptive Statistics

An initial contingency table was created with simple counts of cats according to SWA PA, social system, age, sex, and Q. A table with the positive and negative cases for each infectious agent and each risk factor category was designed, to gain a general appreciation of the distribution of the results (Table S1).

### 2.7. Prevalence

Overall prevalence for the 11 infectious agents (Table S2), as well as prevalences according to the categories of the five independent variables (PA, social system, age, sex, and BCS; Table S3), were calculated with a 95% CI, using R package *binom* [46] and applying the binomial exact method.

### 2.8. Association between “Q” and “Social System”

Biologically, it is not uncommon for domestic cats to live in colonies, whereas wildcats are usually considered solitary. Logistic regression was used to investigate a possible association between Q and social system.

### 2.9. “Q” as a Risk Factor

Univariable analysis was performed to investigate the association between Q and the presence of individual infectious agents using logistic regression models. As inspection of the data suggested the possibility of non-linear relationships between Q and the log odds of positivity, both linear and quadratic models were evaluated. Exploratory analysis also suggested geographic clustering of positivity, so models were also evaluated with a random effect term for SWA PA. Thus, four models (linear/quadratic combined with fixed/random effect) were estimated for each infectious agent. Models were compared with Akaike's information criteria (AIC—a parameter count penalised measure of model fit) and the most parsimonious model within two units of the lowest AIC was selected. The likelihood/probability of infection is presented as the odds ratio (OR). Where quadratic relationships were identified, Q was centred to the mean and scaled by its standard deviation to stabilise estimates of standard errors. Logistic regression models were repeated using the binary variable social system as a predictor. Then variables for age, sex, social system, and BCS were added individually to the models where Q was a significant predictor of infection, to assess if the findings were robust. Wald's test was used to assess the significance of association between predictors and probability of infection and is presented as the probability (p) value. Statistical significance was accepted at *p* < 0.05.

### 2.10. Correlation Analysis

Using R package corrplot, a qualitative correlation plot was created to empirically investigate coinfections and possible associations between infectious agents.

### 2.11. Association between “Pathogen Richness” and “Q”

To explore the relationship between pathogen richness (or coinfections) and positivity, for each of the 11 infectious agents, we calculated the total count of positive results for each individual cat (excluding the outcome infectious agent in its respective model). We added this count as a linear predictor in each of the final models described previously. Additionally, we plotted the total pathogen count for each cat against their hybrid score, Q.

## 3. Results

**Figure 3:**
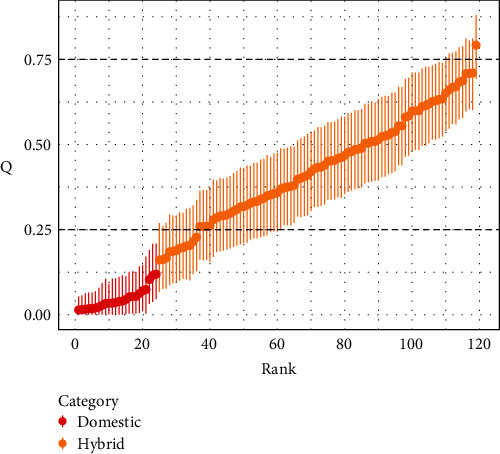
Hybrid scores for all individual cats included in the study. Each cat is given an estimated hybrid score Q by the software STRUCTURE [3] with the limits of the lower and upper boundary of the 90% credibility interval marked with the vertical error bars. The scores have been ranked according to their position in the global dataset. Cats classed as hybrid are orange (LBQ > 0.25, UBQ < 0.75) and those with UBQ ≤ 0.25 are classed as domestic and are presented in red. No cats met the 75% cut-off (LBQ ≥ 0.75) to be classified as wildcats.

A total of 120 free-living Scottish cats were included in the analysis (Table 3). The sex-ratio of sampled cats was fairly even (54F and 65M), however most cats were older than 1 year (*n* = 103), with only 15 cats under 1 year of age. When Q is converted into categories, as defined by Senn et al. [3], there were 24 domestic cats, 96 hybrids, and no wildcats (Figure 3). The majority of cats were from Strathbogie (SB, *n* = 49). Most of the sampled cats were solitary (*n* = 84), compared to those living in the colonies (*n* = 36).

### 3.1. Overall Prevalence

**Figure 4:**
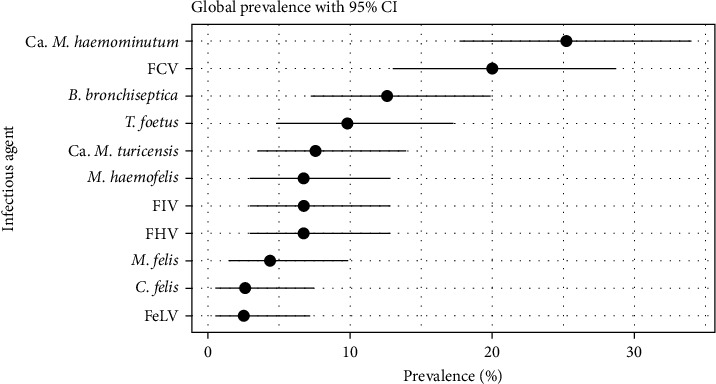
Overall prevalence for the infectious agents included in the study. Error bars represent exact binominal 95% confidence intervals.

Overall prevalence estimates are shown in Figure 4. The lowest prevalence was 2.52% for FeLV (95% CI : 0.52%–7.19%) and the highest 25.2% for *Ca. M. haemominutum* (95% CI : 17.7%–34.0%).

### 3.2. Association between “Q” and “Social System”

**Figure 5:**
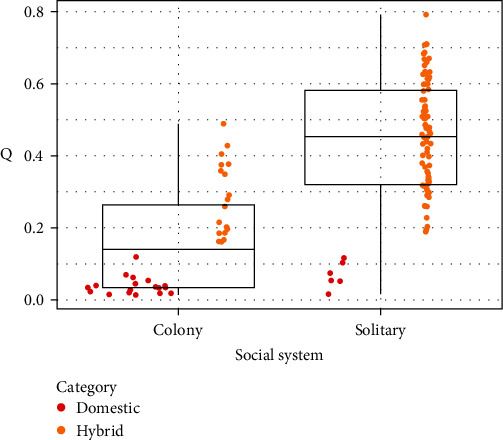
Box plot overlapped with scatter plot displaying the relationship between Q score and social system. Coloured dots represent hybrid scale categories, based on *Q* score value [3]: red-UBQ ≤ 0.25 (domestic); orange-LBQ > 0.25, UBQ < 0.75 (hybrid).

The logistic regression results revealed that Q and social system are highly associated (*p* < 0.001). The lower the Q (i.e., the closer the cat is to “domestic”), the higher the probability of living in a colony (Figure 5).

### 3.3. “Q” as a Risk Factor

**Figure 6:**
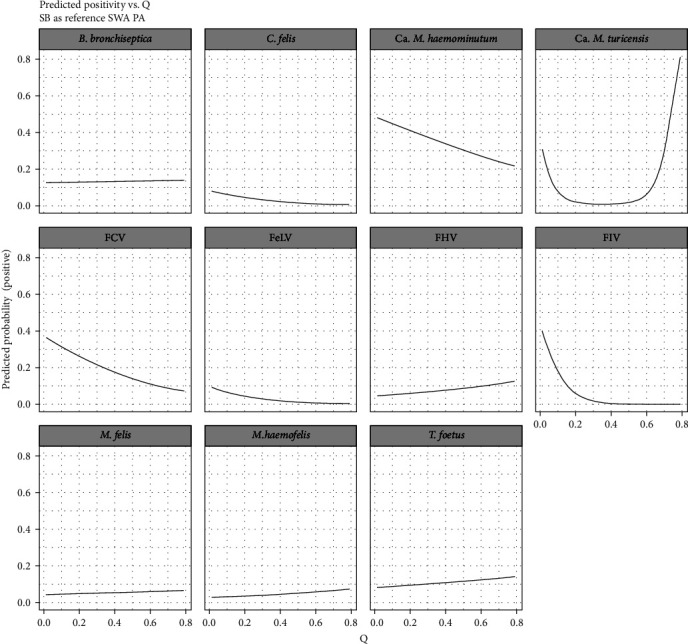
Predicted positivity for each infectious agent vs. Q score. For infectious agents with geographical clustering, prediction is based on Strathbogie (SB) region.

The association between Q and infection with FIV was highly significant (*p*=0.003; Table 4, Figure 6). There was also a statistically significant effect on FCV infection (*p*=0.029). For these two agents, the odds ratio (OR = 4.16 × 10^−6^, 95% CI = 1.12 × 10^−9^−0.016, and OR = 0.077, 95% CI = 0.008–0.772, respectively; Table 4) and predicted positivity (Figure 6) support a negative association between Q and infection; cats with a lower Q (closer to domestic) had a higher probability of infection. For Ca. *M. turicensis*, a significant quadratic association was identified, with Q having a *U*-shaped relationship, suggesting that cats at both ends of the scale are more likely to be infected.

As expected, similarly to Q, the effect of social system as a factor was statistically significant for FIV (*p*=0.014) and FCV (*p*=0.021) (Table 4, Figure 6). ORs of 0.146 (95% CI = 0.032–0.673) and 0.323 (95% CI = 0.124–0.846), respectively, revealed a significantly lower risk for solitary cats to be infected. However, social system did not present a statistically significant effect on Ca. *M. turicensis* infection (*p*=0.412).

Effects of the inclusion of other variables (social system, age, sex, and BCS) on the models where a significant association with Q was observed, are shown in Table 4.

None of the infectious agents presented a statistically significant higher probability of infection in cats with a higher Q (with the exception of Ca. *M. turicensis* described above). However, *B. bronchiseptica*, FHV, *M. haemofelis*, *M. felis*, and *T. foetus*, tended to be slightly more prevalent in cats towards the upper end of the hybrid scale (Figure 6).

### 3.4. Correlation Analysis

**Figure 7:**
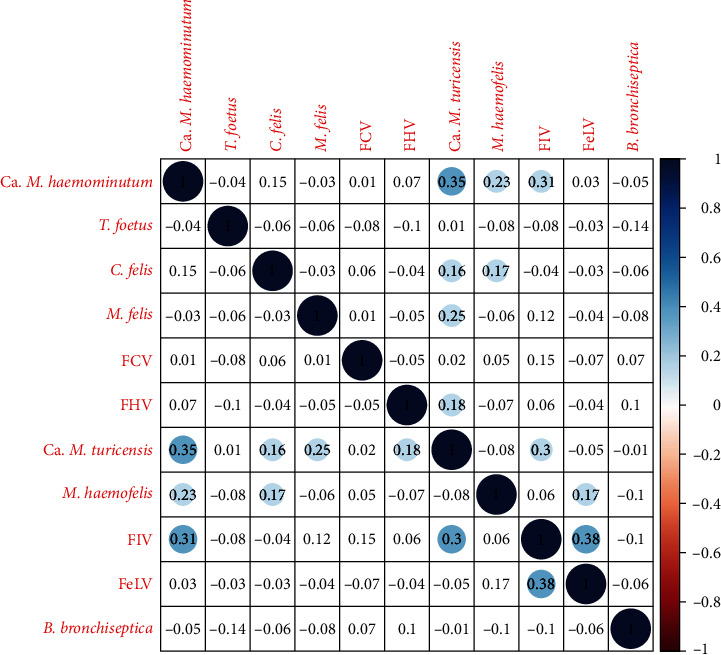
Correlation plot for all the feline infectious agents analysed. Blue colour/positive results represent a positive correlation and red colour/negative results a negative correlation.

The correlation analysis (Figure 7) revealed several strong positive associations: Ca. *M. haemomintum* with Ca. *M. turicensis*, *M. haemofelis* and FIV; Ca. *M. turicensis* with FIV, and *M. felis*; FIV with FeLV. No strong negative correlations were observed.

### 3.5. Association between “Pathogen Richness” and “Q”

**Figure 8:**
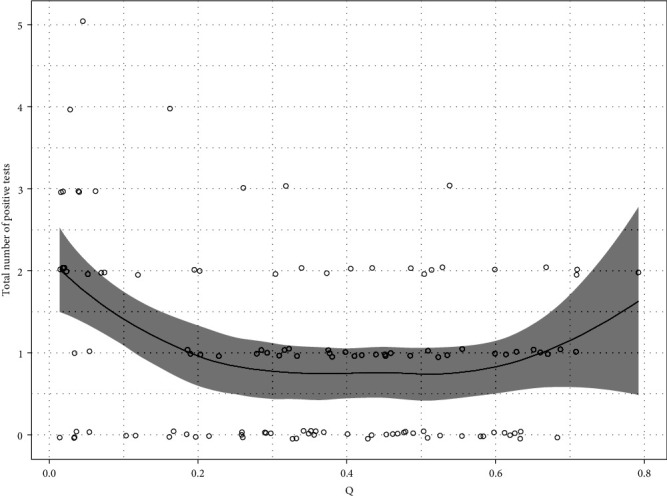
Pathogen richness (total pathogen count) plotted against hybrid score (Q) for each individual cat. The grey line is a LOESS smoother and the grey band its 95% confidence interval. Points are slightly vertically jittered to separate similar values.

The pathogen richness measure (“other” pathogen count) was a significant predictor of test positivity for Candidatus Mycoplasma haemominutum (*p*=0.019) and Candidatus Mycoplasma turicensis (*p*=0.024), with pathogen richness positively associated with test positivity for the respective agent. For these two infections, the inclusion of the pathogen richness measure did not substantively alter the estimates of the relationship between Q and positivity.  Figure 8 shows pathogen richness (total pathogen count) plotted against the hybrid score, Q. There is a suggestion of slightly higher pathogen richness for very low and possibly very high-Q scores.

## 4. Discussion

This study confirms the presence of 11 infectious agents circulating in the free-living cat population in Scotland, which consists of a genetic continuum between *F. silvestris* and *F. catus*. To the authors' knowledge this is the first *in situ* study investigating the infectious disease status of a hybridised wild–domestic species population, where the hybrid score of each individual was assessed using genetic techniques and evaluated as a possible risk factor.

### 4.1. Overall Prevalence in the Context of Previous Studies

A study of feline retroviruses in the UK estimated a prevalence of 9.5% for FIV and 2.3% for FeLV, in domestic cats presented among two animal shelters [47]. We found a similar prevalence for FeLV (2.52%, 95% CI = 0.52%–7.19%), but a slightly lower prevalence for FIV (6.72%, 95% CI = 2.95%–12.8%). This difference could be due to several factors, including geographical variation in the distribution of infection, differences in the diagnostic tests and various characteristics of the populations sampled (e.g., age or social system). Two studies have demonstrated exposure to FIV in *F. silvestris* populations [14, 16], but none have demonstrated infection in the mainland Europe. Two cases of FIV infection (detected by PCR) were reported in Scotland, but these were domestic–wildcat hybrids [48]. Thus, it is still undetermined whether FIV can infect (and cause disease in) genetically distinct wildcats. In contrast, FeLV infection has been detected in several wildcat studies [12–15, 21, 23, 49], including Scotland [18, 50]. However, the genetics of the Scottish cats is unknown and they were probably hybridised. Evidence from France has indicated a possible negative effect of FeLV on wildcat BCS [12, 14], suggesting a potential impact on individual health. Variation in BCS throughout the year, due to physiological and environmental conditions (weather, prey availability etc.), should be considered in the future studies. Furthermore, BCS in the present study was assessed by people with diverse levels of experience, training, and inherent biases (SWA veterinarians, project officers, and volunteers, including members of the public), making it quite subjective, even though it followed a standardised monitoring system [41]. Futures studies should take this into account and optimise BCS assessment. Regardless, the potential impact of FeLV on wild felid populations was demonstrated by the epidemic in a small Iberian lynx (*Lynx pardinus*) population, which caused high morbidity and mortality [51].

Regarding the feline infectious respiratory agents, FCV presented the second highest prevalence (20%, 95% CI : 13.0%–28.7%), followed by *B. bronchiseptica* (12.6%, 95% CI : 7.23%–19.9%). Prevalences for FHV (6.72%, 95% CI : 2.95%–12.8%) and, especially, *C. felis* (2.61%, 95% CI : 0.54%–7.43%) and *M. felis* (4.35%, 95% CI : 1.43%–9.85%) were quite low, which limited the statistical power of the analysis. FCV and FHV prevalences were consistent with the previous studies in European wildcat populations [13, 18, 23], where FCV was considerably more prevalent than FHV. However, FHV low prevalence may reflect the intermittent nature of viral shedding during latency [52]. FCV infection and FHV exposure were detected in the wildcats in Scotland (although, as mentioned previously, the genetics of these cats are unknown), with a prevalence of 26% and seroprevalence of 16%, respectively [18]. This is also consistent with epidemiological studies in the domestic cats (summarised by Cohn [31] and Chandler et al. [53]). It should be noted that most of the trapping in the present study was carried out during winter, when kittens born in the previous spring would have been close to 1 year old. This may have biased the sample away from very young cats. Since domestic kittens show considerably higher morbidity and mortality due to the respiratory infections, as well as retroviruses [53], it is recommended that future studies include a larger sample of younger cats, particularly if trying to assess the role of disease in wild population declines.

Ca. *M. haemominutum* was the infection with the highest overall prevalence (25.2%, 95% CI : 17.7%–34%). The other two haemoplasmas, *M. haemofelis* and Ca. *M. turicensis*, had a prevalence of 6.72% (95% CI : 2.95%–12.8%) and 7.56% (95% CI : 3.52%–13.19%), respectively. These results are consistent with the previous surveys in domestic cats, in the UK and other countries, where Ca. *M. haemominutum* showed a considerably higher prevalence, followed by Ca. *M. turicensis*, which was slightly more prevalent than *M. haemofelis* [54], although this last aspect was not consistent in the other studies [38, 55–60]. Interestingly, a survey of the three haemoplasmas in wild felids, including 31 European wildcats from France [15], found Ca. *M. turicensis* to be the most prevalent (36%), compared to Ca. *M. haemominutum* (19%) and *M. haemofelis* (3%). Several aspects could have influenced this result, such as geographical variations and sample population differences, but a higher susceptibility of *F. silvestris* to Ca. *M. turicensis* cannot be excluded. Curiously, the highest scoring hybrid included in the present study (Q = 0.792, the genetically closest to a wildcat) tested positive for *M. turicensis*, but none of the other haemoplasmas. This cat was also positive for *M. felis*, which showed a relatively high-positive correlation with *M. turicensis* (Figure 7). Coinfections between haemoplasmas and other pathogens, particularly retroviruses are common [15, 36, 61]. This is also supported by our analysis of pathogen richness, which revealed a positive association with test positivity for Ca. *M. turicensis* and Ca. *M. haemominutum*. All five cats testing positive for haemoplasmas in Morvern presented the same coinfection with Ca. *M. haemominutum* and *M. haemofelis*. This could suggest a specific transmission mode in that PA, e.g., a common vector able to transmit both species. Being a peninsula, the free-living cat population on Morvern may be epidemiologically more isolated.

Figure 7 shows a high level of coinfection between FIV and two of the haemoplasmas–Ca. *M. haemominutum* and Ca. *M. turicensis*. In domestic cats, studies suggest that retrovirus-positive cats may be at higher risk of infection with haemoplasmas and this coinfection may exacerbate the severity of disease [36, 62], likely due to retrovirus-mediated immunosuppression. Furthermore, haemoplasmas have been detected in saliva, salivary glands, gingiva, and claw beds of domestic cats [37, 63, 64], suggesting a similar transmission to FIV, through fighting and biting.

The prevalence of 8.8% (95% CI : 4.8%–17.3%) for *T. foetus* is lower than the 14% found in a previous study conducted on UK domestic cats [65]. However, this study included samples from cats with diarrhoea, specifically submitted for *T. foetus* PCR. Furthermore, the cats were all owned, with variable indoor and outdoor lifestyles and living in different cat density situations. To the authors' knowledge, the present study is the first to assess *T. foetus* prevalence in free-living cats in the UK, specifically in Scotland. In other countries, *T. foetus* prevalence has been described as ranging from 2% to 59% (summarised by Gookin et al. [66]) and feline trichomonosis is recognised as an emerging cause of diarrhoea in domestic cats, with worldwide distribution [36].

### 4.2. Infection Status across the Hybrid Scale

For 8 of the 11 infectious agents investigated, namely FeLV, FHV, *C. felis*, *B. bronchiseptica*, *M. felis*, *M. haemofelis*, Ca. *M. haemominutum*, and *T. foetus*, the results demonstrated no significant association between infection probability and Q, supporting our hypothesis that the hybrid swarm may be functioning as a single epidemiological unit.

Infection with FIV, FCV, and Ca. *M. turicensis* was, however, significantly associated with Q. For FIV and FCV, lower scoring cats (closer to domestic) were more likely to test positive. Interestingly, for Ca. *M. turicensis*, cats at both ends of Q (domestic and wildcat ends of the scale), showed a higher probability of infection (Figure 6). As predicted, the analysis confirmed an association between Q and social system, with a lower Q corresponding to a higher likelihood of living in a colony (Figure 5). The modelling of social system as a risk factor resulted in significance for FIV and FCV, similarly to Q, but not for Ca. *M. turicensis* (Table 4). The increased risk of colony cats (and, consequently, cats closer to domestic) being infected with FIV could be explained by a greater probability of these cats coming into contact with infected individuals, due to a higher population density [67]. Considering that transmission of FCV occurs through respiratory, nasal and ocular secretions, requiring a close contact between individuals, it is not surprising that cats living in colonies (and with a lower Q) have a higher probability of infection [53, 67]. Ca. *M. turicencis* infection seems to be more prevalent in cats at both ends of Q (Figure 6), suggesting that mid-scale hybrids are less likely to be infected. This is an interesting and thought-provoking result. Increased fitness in hybrids has been shown in several mammal species [68–71]. Despite the obvious threat posed by introgression to wildcat genetic integrity, we may need to consider whether hybrids can play a role in the conservation of the species, particularly in a swarm population. Currently, the conservation value and legal protection of hybrids is unclear. The fact that Ca. *M. turicensis* infection was significantly associated with Q, but not social system, suggests a potential genetic effect, rather than behavioural or social. However, we must interpret this carefully, as Q is an efficient measure of hybridisation, but does not reflect immunogenetics. Studies into this aspect would be valuable.

When individually adding social system, age group, sex, and BCS to the significant models (FIV, FCV, and Ca. *M. turicensis*), the significance was lost for FCV when social system was included. This suggests that Q may only be significant for FCV infection, since it represents a proxy for social system. As discussed in the previous section, given the close contact transmission mode of FCV, this is unsurprising.

In terms of association between Q and the presence of coinfections, our analysis suggests a slightly higher pathogen richness for very low and possibly very high-hybrid scores (Figure 8). This is an interesting result as it resembles the *U*-shaped association seen between Q score and Ca. *M. turicensis* positivity. However, inferences about the relationship between coinfections and Q score, as well as pathogen richness and individual infectious agent positivity, are challenging in a cross-sectional study such as ours. This relevant area of research would benefit from further studies, particularly longitudinal monitoring of the individuals' infection status over time. This would, not only allow a better understanding of coinfections from a temporal and causal perspective, but also of the potential impact they may have on an individual and population levels.

### 4.3. Infections More Prevalent in Higher Q Scoring Cats

No statistically significant results were obtained to suggest a higher probability of infection in higher genetic scoring cats (with the exception of Ca. *M. turicensis* discussed above). However, although the difference was not significant, *B. bronchiseptica*, FHV, *M. haemofelis*, *M. felis*, and *T. foetus* tended to be more prevalent in cats towards the upper end of the hybrid scale (Figure 6).

It is unclear why FHV and *M. felis* infection may be more prevalent in cats towards the wildcat end of the scale, since their transmission routes are similar to FCV. However, their prevalence (6.72% and 4.35%, respectively) was significantly lower, which likely weakened the statistical analysis. Similarly, the relevance for *M. haemofelis* is uncertain.

*B. bronchiseptica* had the third higher prevalence (12.6%) and, unlike the other respiratory pathogens, it can infect other species, such as dogs and rabbits [53]. It is possible that different sympatric species, inhabiting the different PAs, could play a role in the epidemiology of this disease. Furthermore, the result could suggest that wildcats/high-scoring hybrids may be epidemiologically involved in certain infection cycles where domestic cats/low-scoring hybrids do not play a main role. It should be considered that a large part of the felid community in each PA was not sampled, particularly owned domestic cats who may have outdoor access and potentially transmit infectious agents to in-contact free-living cats.

Some studies have shown that pure-breed domestic cats appear to be at increased risk for *T. foetus*, suggesting a genetic predisposition to infection [65, 66]. However, the samples tended to be overrepresented with pure-breed cats, and the higher rate of infection in these groups may instead be a result of high-risk husbandry conditions (e.g., high densities and poor hygiene conditions). Xenoulis et al. [72] found that the majority of cats infected with *T. foetus* in the UK were domestic shorthair (DSH) cats, contradicting the higher infection tendency in pedigrees. This may be significant in terms of wildcat health, as they are more likely to encounter DSH than pure-breed cats, since the latter tend to be kept indoors.

Although there is limited statistically significant evidence for cats with a higher Q being at a higher risk of infection, the small sample size and the fact that there were no genotypical wildcats in the study need to be considered. We should be wary of over-interpretating the available data, as the results presented here do not allow us to exclude a possible higher susceptibility of wildcats to common feline pathogens. On the other hand, for 8 of the 11 pathogens investigated, the absence of statistical significance in the distribution of infection across the hybrid scale supports our hypothesis that the Scottish hybrid swarm may be acting as a single epidemiological unit.

### 4.4. Gaps in Knowledge and Future Directions

Past studies across the European wildcat range varied in terms of infectious agents investigated and methodologies applied. This makes them difficult to compare and weakens the potential benefits of a structured disease surveillance system across Europe. Furthermore, the morbidity and mortality impact of feline infectious agents and the real risks they may pose for wildcat conservation (i.e., if they have population-limiting consequences) are still unclear. It is also unknown whether these infections can be maintained in wildcat populations, independently of a domestic cat/hybrid reservoir. Other infectious agents, specific to wildcats or transmitted by other sympatric species (particularly domestic and wild carnivores, and prey species such as wild rabbit and rodents), may also affect wildcat health. In addition, anthropogenic impacts, such as human encroachment and habitat fragmentation, may increase the risk of non-infectious diseases, particularly trauma due to road traffic accidents and exposure to environmental toxins [73].

Future research and development of a disease surveillance programme across the European wildcat range should adopt a comprehensive approach. Alongside detection of infection, associated clinical, genetic (including immunogenetics), ecological, behavioural, and histopathological data is necessary to better understand the epidemiology of these diseases and to establish their pathogenicity in wildcats. Sympatric domestic cats and hybrids should be included in the surveillance and, whenever possible, longitudinal sampling should be performed. Applying this multi-disciplinary approach to a systematic analysis of European wildcat health is essential to generate sound scientific evidence, critical to the development of efficient conservation measures. Now that ongoing wildcat population reinforcements are taking place in Scotland, this becomes particularly crucial. Health monitoring and disease surveillance, facilitated by epidemiological studies, become pivotal in strategically shaping and safeguarding these translocation actions.

Finally, our epidemiological approach to a wildlife–domestic species interface starts to bridge a vital gap in the literature, by providing empirical insights into disease transmission dynamics within hybridised populations. Ultimately, this type of research could be applied to inform targeted conservation of other species, particularly endangered ones, where hybridisation and parallel infectious disease transmission are considered as a potential risk.

## 5. Conclusion

This study demonstrates the presence of 11 common feline infectious agents in the free-living cat population of Scotland, which consists of a genetic continuum between *F. silvestris* and *F. catus*. Eight of these agents showed no significant difference in terms of probability of infection across the hybrid scale, suggesting that the Scottish hybrid swarm could constitute a single epidemiological unit, effectively functioning as a reservoir community for these pathogens. Senn et al. [3] exposed a situation where the contemporary free-living cat population contains so many hybrids that they mate with each other and produce more complex hybrids. It is likely that the epidemiology of feline diseases is also becoming increasingly more complex, requiring further investigation, in order to assess the real risks that might impact present and future wildcat conservation efforts. Considering the effects that infectious diseases present for the health, welfare and population dynamics of domestic cats, their presence in the threatened and hybridised population of *F. silvestris* in Scotland, could be population limiting or contribute to local extinction. Comprehensive disease surveillance and risk analysis, in parallel with domestic cat management measures such as those initiated by SWA (vaccination and neutering, public education on responsible cat ownership), will be essential aspects of European wildcat conservation, particularly in areas where hybridisation rates are increasing and anthropogenic factors are prevalent.

## Figures and Tables

**Table 1:** Groups of feline infectious agents included in the study and their pathogenic relevance.

Feline infectious agents category	Pathogenic relevance	Infectious agent	Diagnostic test	Sample collected	References
Retroviruses	Frequent cause of morbidity and mortality in domestic cats worldwide, by inducing immune suppression and increasing vulnerability to secondary or associated diseases [28]. Variable level of pathogenicity described in wild felid species	Feline immunodeficiency virus clade A (FIV)	PCR	Whole blood EDTA	Pinches et al. [29]
		Feline leukaemia virus (FeLV)	PCR	Whole blood EDTA	Pinches et al. [30]

Feline infectious respiratory complex	Significant cause of morbidity in domestic cats, particularly kittens, despite the widespread use of vaccination [31]. Few studies on the significance of respiratory pathogens in wild felids [32]	Feline herpesvirus (FHV)	PCR	Oropharyngeal swab	Helps et al. [33]
		Feline calicivirus (FCV)	RT–PCR	Oropharyngeal swab	Helps et al. [34]
		*Chlamydia felis*	PCR	Conjunctival swab	Helps et al. [33]
		*Mycoplasma felis*	PCR	Conjunctival swab	Not published
		*Bordetella bronchiseptica*	PCR	Oropharyngeal swab	Helps et al. [35]

Feline haemotropic mycoplasmas or haemoplasmas	Potential cause of haemolytic anaemia in domestic cats [36]. Widespread distribution in felid species worldwide [37] leading to growing concern of the potential impact on wild felid conservation	*Mycoplasma haemofelis*	PCR	Whole blood EDTA	Peters et al. [38]
		Candidatus *Mycoplasma haemominutum*	PCR	Whole blood EDTA	Peters et al. [38]
		Candidatus *Mycoplasma turicensis*	PCR	Whole blood EDTA	Peters et al. [38]

Infectious gastroenteritis	Considered one of the most common infectious causes of colitis, particularly in young cats [36]. Worldwide distribution. To the authors' knowledge, there are no studies investigating *T. foetus* in wild felids	*Tritrichomonas foetus*	PCR	Rectal swab	Not published

*Note*: samples collected from live free-living cats, during the trap-neuter-vaccinate-release and wildcat trapping schemes, and diagnostic tests conducted for each infectious agent screening.

**Table 2:** Variables extracted from the SWA database and alterations made prior to analysis.

Original SWA variable	Original description and categories, according to SWA dictionary	Type of variable	Adjusted variable name	Adjusted variable description and categories
Q score	Numeric variable (0–1), based on a 35 SNP genetic marker test, representing the estimated probability of the cat being a wildcat, or “the proportion of wildcat”	Continuous	Unaltered	Unaltered

SWA priority area (SWA PA)	Priority area where the trapping of each individual cat took place: (i) “Morvern” (MV), (ii) “Strathpeffer” (SP), (iii) “Northern Strathspey” (SS), (iv) “Strathbogie” (SB), (v) “Angus Glens” (AG), (vi) “Strathavon” (SA) A seventh category, “non-PA,” included three cats trapped outside the defined PAs	Nominal	Unaltered	“Strathavon (SA)” (with only one cat) was included in “Strathspey (SS),” based on the geographical proximity of these two areas; the three “non-PA” cats were included in “Strathbogie (SB),” since, geographically, these cats locations completely overlap or are very close to the limits of SB

Colony	Classification based on whether the cat was known or expected to live in a group or not. Included three categories: (i) “No” (the cat was caught at a site where a group of cats was not known or expected to live), (ii) “< = 5 cats” (the cat was caught at a site with five or less cats), (iii) “>5 cats” (the cat was caught at a site with more than five cats, except if a queen was caught with more than four kittens)	Nominal	Social system	New categories: (i) “Colony” (categories “< = 5 cats” and “>5 cats” combined into a single category), (ii) “Solitary” (renamed category “No”)

Age	Visual estimate of the cat's age, based on size, dentition and, occasionally, knowledge from previous surveys; included three categories: (i) “Kitten” (cat younger than 16 weeks old), (ii) “Juvenile” (cat between sixteen weeks and 1 year old), (iii) “Adult” (cat older than 1 year old)	Nominal	Unaltered	New categories: (i) “<1 year” (categories “Kitten” and “Juvenile” combined into a single group), (ii) “> = 1 year” (renamed category “Adult”)

Molecular sex	Sex of the cat as determined by a genetic sex-marker: (i) “Female” (genetic analysis determined cat is a female), (ii) “Male” (genetic analysis determined cat is a male)	Binary	Sex	Unaltered

Body condition score (BCS)	The BCS for each cat was visually assessed and classified according to an ascending one-to-five scale, with one being considered “emaciated,” three “ideal” and five “obese” [41]	Nominal	BCS	Two categories were created for this variable: (i) “<3” (underweight), (ii) “> = 3” (normal to overweight)

*Note*: original variables and categories are described according to a “dictionary” provided by SWA.

**Table 3:** Summary of the distribution of the cats included in the study, according to SWA priority area, age, sex, and Q score category (based on Q score value, according to Senn et al. [3].

SWA PA	Total cats	Age group			Sex			Q score category			Social system	
		≥ = 1 year	<1 year	NA	Female	Male	NA	Domestic	Hybrid	Wildcat	Solitary	Colony
AG	33	23	8	2	22	11		0	33	0	33	0
MV	10	9	1		3	7		2	8	0	4	6
SB	49	48	1		15	33	1	18	31	0	28	21
SP	20	17	3		10	10		3	17	0	16	4
SS	8	6	2		4	4		1	7	0	3	5
Total	120	103	15	2	54	65	1	24	96	0	84	36

*Note*: UBQ ≤ 0.25–domestic; LBQ > 0.25, UBQ < 0.75–hybrid; LBQ ≥ 0.75–wildcat), and social system. NA: not assessed at the time of capture (age), genetic test failed (sex), or unknown. AG–, Angus glens; MV–, Morvern; SB, Strathbogie; SP, Strathpeffer; SS, Strathspey.

**Table 4:** Summary of results for logistic regression models of infection.

Risk factor		FIV	FeLV	FCV	FHV	*C. felis*	*B. bronchiseptica*	*M. felis*	*M. haemofelis*	Ca. *M. haemominutum*	Ca. *M. turicensis*	*T. foetus*
Q score	OR (95% CI)	4.16 × 10^−6^ (1.12 × 10^−9^–0.016)	0.013 (4.33 × 10^−5^–3.749)	0.077 (0.008–0.772)	4.077 (0.135–123.210)	0.038 (1.86 × 10^−4^– 7.897)	1.158 (0.090– 14.958)	1.860 (0.032–107.570)	3.734 (0.062–226.378) +RE	0.214 (0.023–1.979) +RE	Q: 0.994 (0.596–1.659) Q2 ^*∗*^: 4.022 (1.756–9.209) ^*∗*^scaled Q	2.171 (0.104–45.117)
	*p* Value	**0.003** ^*∗*^ ^*∗*^	0.133	**0.029** ^*∗*^	0.419	0.230	0.911	0.764	0.529	0.174	0.983, **0.001** ^*∗*^ ^*∗*^ ^*∗*^	0.617

Social system (ref. colony)	OR (95% CI)	0.146 (0.032– 0.673)	3.214 (0.156– 66.336)	0.323 (0.124– 0.846)	1.124 (0.243–5.200)	0.235 (0.029–1.890)	1.124 (0.243–5.200)	1.258 (0.185–8.555)	1.764 (0.254–12.238) +RE	1.194 (0.449–3.175) +RE	0.539 (0.123–2.356)	0.867 (0.221–3.396)
	*p* Value	**0.014** ^*∗*^	0.450	**0.021** ^*∗*^	0.881	0.173	0.196	0.814	0.566	0.722	0.412	0.838

Q score (with social system in model)	OR (95% CI)	2.07 × 10^−6^ (0.00–0.02)		0.205 (0.011– 3.854)							1.777 (0.596–5.296) 3.459 (1.590–7.527)	
	*p* Value	**0.005** ^*∗*^ ^*∗*^		0.290							0.302, **0.002** ^*∗*^ ^*∗*^	

Q score (with age group in model)	OR (95% CI)	7.07 × 10^−6^ (2.06 × 10^−9^–0.024)		0.079 (0.007– 0.882)							1.020 (0.613–1.697) 3.655 (1.621–8.239)	
	*p* Value	**0.004** ^*∗*^ ^*∗*^		**0.039** ^*∗*^							0.939, **0.002** ^*∗*^ ^*∗*^	

Q score (with sex in model)	OR (95% CI)	1.60 × 10^−5^ (6.51 × 10^−9^–0.039)		0.123 (0.012–1.251)							1.164 (0.681–1.989) 4.281 (1.798–10.193)	
	*p* Value	**0.006** ^*∗*^ ^*∗*^		0.077							0.578, **0.001** ^*∗*^ ^*∗*^ ^*∗*^	

Q score (with BCS > 3 in model)	OR (95% CI)	1.04 × 10^−5^ (5.52 × 10^−9^–0.020)		0.071 (0.007– 0.685)							1.046 (0.615–1.781) 4.187 (1.790–9.794)	
	*p* Value	**0.003** ^*∗*^ ^*∗*^		**0.022** ^*∗*^							0.867, **0.001** ^*∗*^ ^*∗*^ ^*∗*^	

*Note*: the first two rows report univariable models for Q score and social system. Rows three onwards show results of significant Q score models with addition of social system, age group, sex, and body condition score (BCS). For some infectious agents, the models that included a random effect (associated with clustering) presented a better fit. The results for these are labelled with “+RE”. Results in bold were considered statistically significant:  ^*∗∗∗*^*p* < 0.001;  ^*∗∗*^0.001 < *p* < 0.01;  ^*∗*^0.01 < *p* < 0.05.

## Data Availability

R script is included in the supplementary materials. Dataset and precise spatial coordinates for free-living cats are available from the authors upon reasonable request.

## Supplementary Materials

Supplementary Materials Supplementary materials include the R script for the models used in the statistical analysis. Table S1: counts of positive and negative results for each infectious agent and each risk factor category, as used in the statistical analysis. Table S2: summary of the overall prevalences and CIs (95%) for each infectious agent included in the study. Table S3: prevalences and 95% CIs of each infectious agent, for the individual risk factor categories.
