# Supplementary material for: Epidemiology of a Hybrid Swarm: Evidence of 11 Feline Infectious Agents Circulating in a Population of Sympatric European Wildcat Hybrids and Free-Living Domestic Cats, in Scotland
Source: Transbound Emerg Dis. 2023 Oct 5;2023:6692514. doi: 10.1155/2023/6692514 (PMC12017057; doi:10.1155/2023/6692514)
Supplement: Supplementary Materials — include the R script for the models used in the statistical analysis. Table S1: counts of positive and negative results for each infectious agent and each risk factor category, as used in the statistical analysis. Table S2: summary of the overall prevalences and CIs (95%) for each infectious agent included in the study. Table S3: prevalences and 95% CIs of each infectious agent, for the individual risk factor categories. [file 6692514.f1.pdf]

Supplementary Materials

Table 1: Counts of positive and negative results for each infectious agent and each risk factor category, as used in the statistical analysis. "-" negative results; "+" positive results.

DISEASE RESULTS	PRIORITY AREA					AGE		BCS		SOCIAL SYSTEM		SEX	
	**AG**	**MV**	**SB**	**SP**	**SS**	**<1 year**	**>=1 year**	**<3**	**>=3**	**Solitary**	**Colony**	**Female**	**Male**
**FIV -**	33	10	42	19	7	15	94	33	75	81	30	54	56
**FIV +**	0	0	7	0	1	0	8	5	3	2	6	0	8
**FeLV -**	33	10	47	49	7	15	99	36	77	80	35	52	63
**FeLV +**	0	0	1	1	1	0	3	2	1	3	0	1	2
**FCV -**	23	9	35	16	5	11	76	24	61	67	21	44	43
**FCV +**	8	1	10	2	1	1	20	8	14	11	11	6	16
**FHV -**	32	9	43	20	7	14	95	33	75	78	33	50	60
**FHV +**	1	1	5	0	1	1	7	4	4	6	2	3	5
***C. felis* -**	31	10	44	19	8	14	96	36	73	81	31	50	61
***C. felis* +**	0	0	3	0	0	0	3	0	3	1	2	0	3
***B. bronchiseptica* -**	24	10	43	20	7	12	90	34	67	71	33	46	57
***B. bronchiseptica* +**	9	0	5	0	1	3	12	3	12	13	2	7	8
***M. felis* -**	31	10	42	19	8	13	95	32	75	78	32	48	61
***M. felis* +**	0	0	5	0	0	1	4	4	1	4	1	2	3
***M. haemofelis* -**	32	5	47	19	8	15	95	37	71	78	33	53	57
***M. haemofelis* +**	1	5	2	0	0	0	7	1	7	5	3	1	7
**Ca. *M. haemominutum* -**	33	5	30	15	6	15	72	30	56	65	24	52	36
**Ca. *M. haemominutum* +**	0	5	19	4	2	0	30	8	22	18	12	2	28
**Ca. *M. turicensis* -**	33	10	40	19	8	15	93	35	72	79	31	53	56
**Ca. *M. turicensis* +**	0	0	9	0	0	0	9	3	6	4	5	1	8
***T. foetus* -**	27	8	37	15	5	11	80	27	63	66	26	40	51
***T. foetus* +**	4	1	2	3	0	1	8	1	9	7	3	7	3

Table 2: Summary of the overall prevalences and confidence intervals (95%) for each infectious agent included in the study (LCI – lower confidence interval, UCI – upper confidence interval).

Infectious agent	Positives	tested	Untested	Prevalence (%)	LCI (%)	UCI (%)
**FIV**	8	119	1	6.72	2.95	12.8
**FeLV**	3	119	1	2.52	0.52	7.19
**FCV**	22	110	10	20.00	13.0	28.7
**FHV**	8	119	1	6.72	2.95	12.8
C. felis	3	115	5	2.61	0.54	7.43
B. bronchiseptica	15	119	1	12.6	7.23	19.9
M. felis	5	115	5	4.35	1.43	9.85
M. haemofelis	8	119	1	6.72	2.95	12.8
**Ca. *M. turicensis***	9	119	1	7.56	3.52	13.9
**Ca. *M. haemominutum***	30	119	1	25.2	17.7	34.0
T. foetus	10	102	18	8.80	4.80	17.3

Table 3: Prevalences and 95% CIs of each infectious agent, for the individual risk factor categories.

**Variable**	**PREVALENCE % (95% CI)**		
		**FIV**	**FeLV**
**SWA PA**	AG	0 (0.00-10.58)	0 (0.00-10.58)
	SB	14.29 (5.94-27.24)	2.08 (0.05-11.07)
	SP	0 (0.00-17.65)	5.00 (0.13-25.87)
	SS	12.50 (0.32-52.65)	12.50 (0.32-52.65)
	MV	0 (0.00-30.85)	0 (0.00-30.85)
**Age group**	<1 year	0 (0.00-21.80)	0 (0.00-21.80)
	>=1 year	7.84 (3.45-14.87)	2.94 (0.61-8.36)
**Sex**	Female	0 (0.00-6.60)	1.89 (0.04-10.07)
	Male	12.5 (5.55-23.15)	3.08 (0.37-10.68)
**social system**	Solitary	2.41 (0.29-8.43)	3.61 (0.75-10.20)
	Colony	16.7 (6.37-32.81)	0 (0.00-9.74)
**BCS**	<3	13.16 (4.41-28.09)	5.26 (0.64-17.75)
	>=3	3.85 (0.80-10.83)	1.28 (0.03-6.93)

Table 3 (continued): Prevalences and 95% CIs of each infectious agent, for the individual risk factor categories.

**Variable**		**Prevalence % (95% CI)**		
		**FCV**	**FHV**	***C. felis***
**SWA PA**	AG	25.81 (11.86-44.61)	3.03 (0.08-15.76)	0 (0.00-11.22)
	SB	22.22 (11.20-37.09)	10.42 (3.47-22.66)	6.38 (1.34-17.54)
	SP	11.11 (1.38-34.71)	0 (0.00-16.84)	0 (0.00-17.65)
	SS	16.37 (0.42-64.12)	12.50 (0.32-52.65)	0 (0.00-36.94)
	MV	10.00 (0.25-44.50)	10.00 (0.25-44.50)	0 (0.00-30.85)
**Age group**	<1 year	8.33 (0.21-38.48)	6.67 (0.17-31.95)	0 (0.00-23.16)
	>= 1 year	20.83 (13.21-30.33)	6.86 (2.80-13.63)	3.03 (0.63-8.60)
**Sex**	Female	12.00 (4.53-24.31)	5.66 (1.18-15.66)	0 (0.00-7.11)
	Male	27.12 (16.36-40.27)	7.69 (2.54-17.05)	4.69 (0.98-13.09)
**social system**	Solitary	14.10 (7.26-23.83)	7.14 (2.67-14.90)	1.22 (0.03-6.61)
	Colony	34.38 (18.57-53.19)	5.71 (0.70-19.16)	6.06 (0.74-20.23)
**BCS**	<3	25.00 (11.46-43.40)	10.81 (3.03-25.42)	0 (0.00-9.74)
	>=3	18.67 (10.60-29.33)	5.06 (1.40-12.46)	3.95 (0.82-11.11)

Table 3 (continued): Prevalences and 95% CIs of each infectious agent, for the individual risk factor categories.

**Variable**		**Prevalence % (95% CI)**		
		M. felis	***B. bronchiseptica***	***T. foetus***
**SWA PA**	AG	0 (0.00-11.22)	27.27 (13.30-45.52)	12.90 (3.63-29.83)
	SB	10.64 (3.55-23.10)	10.42 (3.47-22.66)	5.13 (0.63-17.32)
	SP	0 (0.00-17.65)	0 (0.00-16.84)	16.67 (3.58-41.42)
	SS	0 (0.00-36.94)	12.50 (0.32-52.65)	0 (0.00-52.18)
	MV	0 (0.00-30.85)	0 (0.00-30.85)	11.11 (0.28-48.25)
**Age group**	<1 year	7.14 (0.18-33.87)	20.00 (4.33-48.01)	8.33 (0.21-38.48)
	>=1 year	4.04 (1.11-10.02)	11.76 (6.23-19.65)	9.09 (4.00-17.13)
**Sex**	Female	4.00 (0.49-13.71)	13.21 (5.48-25.34)	14.89 (6.20-28.31)
	Male	4.69 (0.98-13.09)	12.31 (5.47-22.82)	5.56 (1.16-15.39)
**social system**	Solitary	4.88 (1.34-12.02)	15.48 (8.51-25.01)	9.59 (3.94-18.76)
	Colony	3.03 (0.08-15.76)	5.71 (0.70-19.16)	10.34 (2.19-27.35)
**BCS**	<3	11.11 (3.11-26.06)	8.11 (1.70-21.91)	3.57 (0.09-18.35)
	>=3	1.32 (0.03-7.11)	15.19 (8.10-25.03)	12.50 (5.88-22.41)

Table 3 (continued): Prevalences and 95% CIs of each infectious agent, for the individual risk factor categories.

**Variable**	**Prevalence % (95% CI)**			
		M. haemofelis	**Ca. *M. haemominutum***	**Ca. *M. turicensis***
**SWA PA**	AG	3.03 (0.01-15.76)	0 (0.00-10.56)	0 (0.00-10.56)
	SB	4.08 (0.50-13.98)	38.78 (25.20-53.76)	18.37 (8.76-32.02)
	SP	0 (0.00-17.65)	21.05 (6.05-45.57)	0 (0.00-17.65)
	SS	0 (0.00-36.94)	25.00 (3.19-65.09)	0 (0.00-36.94)
	MV	50.00 (18.71-81.29)	50.00 (18.71-81.29)	0 (0.00-30.85)
**Age group**	<1 year	0 (0.00-21.80)	0 (0.00-21.80)	0 (0.00-21.80)
	>=1 year	6.86 (2.80-13.63)	29.41 (20.80-39.25)	8.82 (4.11-16.09)
**Sex**	Female	1.85 (0.04-9.89)	3.70 (0.45-12.75)	1.85 (0.04-9.89)
	Male	10.94 (4.51-21.25)	43.75 (31.37-56.72)	12.50 (5.55-23.15)
**social system**	Solitary	6.02 (1.98-13.50)	21.69 (13.39-32.09)	4.82 (1.33-11.88)
	Colony	8.33 (1.75-22.47)	33.33 (18.56-50.97)	13.89 (4.67-29.50)
**BCS**	<3	2.63 (0.07-13.81)	21.05 (9.55-37.32)	7.89 (1.66-21.38)
	>=3	8.97 (3.68-17.62)	28.21 (18.59-39-53)	7.69 (2.88-15.99)

SWA PA – Scottish Wildcat Action priority area; AG – Angus Glens, SB – Strathbogie, SP – Strathpeffer, SS – Strathspey, MV – Morvern; BCS – Body condition score.

R script for models used in the statistical analysis

```{r}
library(tidyverse) # for data manipulation and plotting
library(lme4) # for models
library(here) # makes file location easier
library(janitor) # for cleaning functions
library(broom) # to tidy model outpu
library(broom.mixed) # to tidy random effect models
library(patchwork) # combines plots
library(brglm2)
library(parameters)
library(gt)
```

Functions

Binomial test that fails gracefully if no data.

```{r}
binom.test2 <- possibly(binom.test, otherwise = list(conf.int = c(NA, NA)))

```

Import data

```{r}
wildc_wide <- read_csv(here("data", "wildc.csv"))


wildc <- wildc_wide %>%
  janitor::clean_names() %>%
  pivot_longer(c_m_haemominutum:b_bronchiseptica,
               names_to = "test",
               values_to = "result") %>%
```

````
  # set SB as reference swa_pa
  mutate(swa_pa = fct_relevel(swa_pa, "SB")) %>%
  # drop NA results
  drop_na(result)

```

### Plot regional prevalences

```{r, fig.height=10, fig.width=10}
  wildc %>%
    group_by(test, swa_pa) %>%
    summarise(r = sum(result, na.rm = TRUE),
              n = sum(!is.na(result)),
              prev = mean(r/n),
              lci = binom.test(r, n)$conf.int[[1]],
              uci = binom.test(r, n)$conf.int[[2]]) %>%
    ungroup() %>%
    ggplot() +
    aes(x = swa_pa, y = prev, ymin = lci, ymax = uci) +
    geom_pointrange(shape = 1) +
    facet_wrap(facets = ~ test, scale = "free", ncol = 3) +
    labs(title = "Prevalence by swa_pa/test",
         caption = "with 95% CI",
         x = "Swarm")
```

# Models

### Fit models

```{r}
````

```
# wildc <- wildc %>%
#   mutate(q = scale(q))

mod <- wildc %>%

  # mutate(q = scale(q)) %>%
  drop_na(result, q) %>%
  group_nest(test) %>%
  mutate(fit_1_m = map(data, ~ glmer(result ~ q + (1 | swa_pa),
    family = binomial(), control=glmerControl(optimizer="bobyqa",
optCtrl=list(maxfun=100000)),
    data = .x,
  ))) %>%
  mutate(fit_2_m = map(data, ~ glmer(result ~ q + I(q^2) + (1 | swa_pa),
    family = binomial(), control=glmerControl(optimizer="bobyqa",
optCtrl=list(maxfun=100000)),
    data = .x,
  ))) %>%
  mutate(fit_1_f = map(data, ~ glm(result ~ q,
    family = binomial(), method = "brglmFit",
    data = .x
  ))) %>%
  mutate(fit_2_f = map(data, ~ glm(result ~ q + I(q^2),
    family = binomial(), method = "brglmFit",
    data = .x
  )))

mod1 <- mod %>%
  pivot_longer(contains("fit"), names_to = "model", values_to = "fit") %>%

  mutate(aic = map_dbl(fit, AIC)) %>%
  mutate(singular = map_lgl(fit, performance::check_singularity)) %>%
```

filter(!singular) %>%

group_by(test) %>%

mutate(delta_aic = aic - min(aic)) %>%

filter(aic <= min(aic) + 2) %>%

ungroup() %>%

manually choose simplest models within 2 AIC of minimum from fitted models above

filter(test == "b_bronchiseptica" & model == "fit_1_f" |
test == "c_m_haemominutum" & model == "fit_1_m" |
test == "chlamydophila_felis" & model == "fit_1_f" |
test == "fcv" & model == "fit_1_f" |
test == "fe_lv" & model == "fit_1_f" |
test == "fhv" & model == "fit_1_f" |
test == "fiv" & model == "fit_1_f" |
test == "m_haemofelis" & model == "fit_1_m" |
test == "m_turicensis" & model == "fit_2_f" |
test == "mycoplasma_felis" & model == "fit_1_f" |
test == "tritrichomonas_foetus" & model == "fit_1_f")
```

### Predict probability of positive vs q (using SB swa_pa as reference)

```{r}
mod1 %>%
mutate(pred = map2(mod1$fit, mod1$data, ~predict(.x, newdata = .y %>% mutate(swa_pa = "SB")))) %>%
filter(!str_detect(test, "snap")) |>
unnest(c(data, pred)) %>%
ggplot() +
aes(x = q, y = plogis(pred)) +
geom_line() +
facet_wrap(~ test) +

~~~
  labs(x = "Q",
       y = "predicted prob(positive",
       title = "Predicted positivity vs Q",
       subtitle = "SB as reference swa_pa")

ggsave("predicted_prob_plot.pdf", height = 8, width = 8)

```

```{r}
mod1 %>%
  mutate(pred = map2(mod1$fit, mod1$data, ~predict(.x, newdata = .y %>% mutate(swa_pa =
"SB")))) %>%
  unnest(c(data, pred)) %>%
  filter(test == "m_turicensis") %>%
  ggplot() +
  aes(x = q, y = plogis(pred)) +
  geom_line() +
  facet_wrap(~ test) +
  labs(x = "Q",
       y = "predicted prob(positive",
       title = "Predicted positivity vs Q",
       subtitle = "SB as reference swa_pa")
```

```{r}
# formatting criteria fro sci format
num_low <- 0.001
num_high <- 1000


mod1 %>%
  mutate(model_type = fct_recode(model,
~~~

```
                    "mixed - linear" = "fit_1_m",
                    "mixed - quadratic" = "fit_2_m",
                    "fixed - linear" = "fit_1_f",
                    "fixed - quadratic" = "fit_2_f")) %>%
  mutate(est = map(fit, parameters, exponentiate = TRUE)) %>%
  unnest(est) %>%
  mutate(Term = str_remove_all(Parameter, "I|\\(|\\)")) %>%
  discard(is.list) %>%
  filter(!str_detect(Parameter, "Intercept|SD")) %>%
  select(Disease = test, `Model type` = model_type, Term, OR = Coefficient, CI_low, CI_high,
`P-value` = p) %>%
  mutate(Disease = fct_relevel(Disease, c("fiv", "fe_lv",
                         "fcv", "fhv", "chlamydophila_felis",
                         "b_bronchiseptica", "mycoplasma_felis", "m_haemofelis",
                         "c_m_haemominutum",
                         "m_turicensis", "tritrichomonas_foetus"))) %>%
  arrange(Disease) %>%
  gt::gt() %>%
  gt::fmt_scientific(OR, rows = OR > num_high | OR < num_low) %>%
  gt::fmt_number(OR, decimals = 3, rows = OR <= num_high & OR >= num_low) %>%
  gt::fmt_scientific(CI_low, rows = CI_low > num_high | CI_low < num_low) %>%
  gt::fmt_number(CI_low, decimals = 3, rows = CI_low <= num_high & CI_low >= num_low)
%>%
  gt::fmt_scientific(CI_high, rows = CI_high > num_high | CI_high < num_low) %>%
  gt::fmt_number(CI_high, decimals = 3, rows = CI_high <= num_high & CI_high >=
num_low) %>%
  gt::fmt_number(`P-value`, decimals = 3) %>%
  gt::cols_merge(OR:CI_high, pattern = "{1}, ({2} - {3})")

```

### Individual models

````
```{r}
tabb <- function(x){
x %>%
  parameters::parameters(exponentiate = TRUE) %>%
  mutate(Term = str_remove_all(Parameter, "I|\\(|\\)")) %>%
  select(Term, OR = Coefficient, CI_low, CI_high, `P-value` = p) %>%
  gt::gt() %>%
  gt::fmt_scientific(OR, rows = OR > num_high | OR < num_low) %>%
  gt::fmt_number(OR, decimals = 3, rows = OR <= num_high & OR >= num_low) %>%
  gt::fmt_scientific(CI_low, rows = CI_low > num_high | CI_low < num_low) %>%
  gt::fmt_number(CI_low, decimals = 3, rows = CI_low <= num_high & CI_low >= num_low) %>%
  gt::fmt_scientific(CI_high, rows = CI_high > num_high | CI_high < num_low) %>%
  gt::fmt_number(CI_high, decimals = 3, rows = CI_high <= num_high & CI_high >= num_low) %>%
  gt::fmt_number(`P-value`, decimals = 3) %>%
  gt::cols_merge(OR:CI_high, pattern = "{1}, ({2} - {3})")
}
```
````

FCV

````
```{r}
wildc %>%
  filter(test == "fcv") %>%
  glm(result ~ q + mol_sex,
    family = binomial(), method = "brglmFit",
    data = .) %>%
  tabb()
```
````

````
```{r}
wildc %>%
````
````

````
  filter(test == "fcv") %>%
  glm(result ~ q + factor(bcs>3),
    family = binomial(), method = "brglmFit",
    data = .) %>%
tabb()
```

```{r}
wildc %>%
  filter(test == "fcv") %>%
  glm(result ~ q + age,
    family = binomial(), method = "brglmFit",
    data = .) %>%
  tabb()
```

```{r}
wildc %>%
  filter(test == "fcv") %>%
  glm(result ~ q + social_sys,
    family = binomial(), method = "brglmFit",
    data = .) %>%
  tabb()
```

### FIV

```{r}
wildc %>%
  filter(test == "fiv") %>%
  glm(result ~ q + social_sys,
    family = binomial(), method = "brglmFit",
````

````
    data = .) %>%
  tabb()
```

```{r}
wildc %>%
  filter(test == "fiv") %>%
  glm(result ~ q + mol_sex,
    family = binomial(), method = "brglmFit",
    data = .) %>%
  tabb()
```

```{r}
wildc %>%
  filter(test == "fiv") %>%
  glm(result ~ q + factor(bcs>3),
    family = binomial(), method = "brglmFit",
    data = .) %>%
tabb()
```

```{r}
wildc %>%
  filter(test == "fiv") %>%
  glm(result ~ q + age,
    family = binomial(), method = "brglmFit",
    data = .) %>%
tabb()
```

### m_turicensis
````
````

````
```{r}
wildc %>%
  filter(test == "m_turicensis") %>%
  glmer(result ~ q + I(q^2) + (1 | swa_pa) + mol_sex,
    family = binomial(), control=glmerControl(optimizer="bobyqa", optCtrl=list(maxfun=100000)),
    data = .) %>%
    tabb()

```

```{r}
wildc %>%
  filter(test == "m_turicensis") %>%
  glmer(result ~ q + I(q^2) + (1 | swa_pa) + factor(bcs>3),
    family = binomial(), control=glmerControl(optimizer="bobyqa", optCtrl=list(maxfun=100000)),
    data = .) %>%
    tabb()

```

```{r}
wildc %>%
  filter(test == "m_turicensis") %>%
  glmer(result ~ q + I(q^2) + (1 | swa_pa) + age,
    family = binomial(), control=glmerControl(optimizer="bobyqa", optCtrl=list(maxfun=100000)),
    data = .) %>%
    parameters::parameters(exponentiate = TRUE)

```
````

```{r}
wildc %>%
  filter(test == "m_turicensis") %>%
  glmer(result ~ q + I(q^2) + (1 | swa_pa) + social_sys,
    family = binomial(), control=glmerControl(optimizer="bobyqa",
optCtrl=list(maxfun=100000)),
    data = .) %>%
    parameters::parameters(exponentiate = TRUE)

```

mycoplasma_felis

```{r}
wildc %>%
  filter(test == "mycoplasma_felis") %>%
  glm(result ~ q + mol_sex,
    family = binomial(), method = "brglmFit",
    data = .) %>%
  parameters::parameters(exponentiate = TRUE)
```

```{r}
wildc %>%
  filter(test == "mycoplasma_felis") %>%
  glm(result ~ q + factor(bcs>3),
    family = binomial(), method = "brglmFit",
    data = .) %>%
  parameters::parameters(exponentiate = TRUE)
```

```{r}
wildc %>%
  filter(test == "mycoplasma_felis") %>%
  glm(result ~ q + age,
    family = binomial(), method = "brglmFit",
    data = .) %>%
  parameters::parameters(exponentiate = TRUE)
```

```{r}
wildc %>%
  filter(test == "mycoplasma_felis") %>%
  glm(result ~ q + social_sys,
    family = binomial(), method = "brglmFit",
    data = .) %>%
  parameters::parameters(exponentiate = TRUE)
```

m_haemofelis

```{r}
wildc %>%
  filter(test == "m_haemofelis") %>%
  glm(result ~ q + mol_sex,
    family = binomial(), method = "brglmFit",
    data = .) %>%
  parameters::parameters(exponentiate = TRUE)
```

```{r}
wildc %>%
  filter(test == "m_haemofelis") %>%
```

  glm(result ~ q + factor(bcs>3),
    family = binomial(), method = "brglmFit",
    data = .) %>%
  parameters::parameters(exponentiate = TRUE)
```

```{r}
wildc %>%
  filter(test == "m_haemofelis") %>%
  glm(result ~ q + age,
    family = binomial(), method = "brglmFit",
    data = .) %>%
  parameters::parameters(exponentiate = TRUE)
```

```{r}
wildc %>%
  filter(test == "m_haemofelis") %>%
  glm(result ~ q + social_sys,
    family = binomial(), method = "brglmFit",
    data = .) %>%
  parameters::parameters(exponentiate = TRUE)
```

Other models

```{r}
wildc %>%
  filter(test == "c_m_haemominutum") %>%
  glm(result ~ q + age,
    family = binomial(), method = "brglmFit",
    data = .) %>%

~~~~
  parameters::parameters(exponentiate = TRUE)
```

Check c_m_haemominutum model

```{r}
lmtest::lrtest(
  wildc %>%
    drop_na(q) %>%
  filter(test == "c_m_haemominutum") %>%
  glm(result ~ 1 + swa_pa,
      family = binomial(),
    data = .),
  wildc %>%
  drop_na(q) %>%
  filter(test == "c_m_haemominutum") %>%
  glm(result ~ q + I(q^2) + swa_pa,
      family = binomial(),
    data = .)
)

```
```{r}
wildc %>%
  filter(test == "fcv") %>%
  glm(result  ~ q,
    family = binomial(),
    data = .) %>%
  parameters::parameters(exponentiate = TRUE)
```

```{r}
~~~~

```
wildc %>%
  filter(test == "fcv") %>%
  # filter(social_sys == "Solitary") %>%
  glm(result  ~ q,
    family = binomial(),
    data = .) %>%
  parameters::parameters(exponentiate = TRUE)
```

Social system models

```{r}
wildc %>%

  # we need to scale q to have model run
  drop_na(result, q) %>%
  group_nest(test) %>%
  mutate(fit_1_m = map(data, ~ glmer(result ~ social_sys + (1 | swa_pa),
    family = binomial(), control=glmerControl(optimizer="bobyqa",
optCtrl=list(maxfun=100000)),
    data = .x,
  ))) %>%
  mutate(fit_1_f = map(data, ~ glm(result ~ social_sys,
    family = binomial(), method = "brglmFit",
    data = .x
  ))) %>%

  pivot_longer(contains("fit"), names_to = "model", values_to = "fit") %>%

  mutate(aic = map_dbl(fit, AIC)) %>%
  mutate(singular = map_lgl(fit, performance::check_singularity)) %>%
  filter(!singular) %>%
  group_by(test) %>%
```

```
  mutate(delta_aic = aic - min(aic)) %>%
  filter(aic <= min(aic) + 2) %>%
  ungroup() %>%

   filter(test == "b_bronchiseptica" & model == "fit_1_f" |
         test == "c_m_haemominutum" & model == "fit_1_m" |
         test == "chlamydophila_felis" & model == "fit_1_f" |
         test == "fcv" & model == "fit_1_f" |
         test == "fe_lv" & model == "fit_1_f" |
         test == "fhv" & model == "fit_1_f" |
         test == "fiv" & model == "fit_1_f" |
         test == "m_haemofelis" & model == "fit_1_m" |
         test == "m_turicensis" & model == "fit_1_m" |
         test == "mycoplasma_felis" & model == "fit_1_f" |
         test == "tritrichomonas_foetus" & model == "fit_1_f") %>%

  mutate(model_type = fct_recode(model,
                              "mixed - linear" = "fit_1_m",
                              "mixed - quadratic" = "fit_2_m",
                              "fixed - linear" = "fit_1_f",
                              "fixed - quadratic" = "fit_2_f")) %>%
  mutate(est = map(fit, parameters, exponentiate = TRUE)) %>%
  unnest(est) %>%
  mutate(Term = str_remove_all(Parameter, "I|\\(|\\)")) %>%
  discard(is.list) %>%
  filter(!str_detect(Parameter, "Intercept|SD")) %>%
  select(Disease = test, `Model type` = model_type, aic, Term, OR = Coefficient, CI_low,
CI_high, `P-value` = p) %>%
  mutate(Disease = fct_relevel(Disease, c("fiv", "fe_lv",
                                   "fcv", "fhv", "chlamydophila_felis",
                                   "b_bronchiseptica", "mycoplasma_felis", "m_haemofelis",
                                   "c_m_haemominutum",
                                   "m_turicensis", "tritrichomonas_foetus"))) %>%
```

~~~
  arrange(Disease) %>%
  gt::gt() %>%
  gt::fmt_scientific(OR, rows = OR > num_high | OR < num_low) %>%
  gt::fmt_number(OR, decimals = 3, rows = OR <= num_high & OR >= num_low) %>%
  gt::fmt_scientific(CI_low, rows = CI_low > num_high | CI_low < num_low) %>%
  gt::fmt_number(CI_low, decimals = 3, rows = CI_low <= num_high & CI_low >= num_low) %>%
  gt::fmt_scientific(CI_high, rows = CI_high > num_high | CI_high < num_low) %>%
  gt::fmt_number(CI_high, decimals = 3, rows = CI_high <= num_high & CI_high >= num_low) %>%
  gt::fmt_number(`P-value`, decimals = 3) %>%
  gt::cols_merge(OR:CI_high, pattern = "{1}, ({2} - {3})")
```
~~~

Use scaled Q for the quadratic

~~~
```{r}
wildc %>%
  mutate(q = scale(q)) %>%
  filter(test == "m_turicensis") %>%
  glm(result ~ q + I(q^2),
    family = binomial(), method = "brglmFit",
    data = .) %>%
    tabb()
```
~~~

~~~
```{r}
wildc %>%
  mutate(q = scale(q)) %>%
  filter(test == "m_turicensis") %>%
  glm(result ~ q + I(q^2) + social_sys,
    family = binomial(), method = "brglmFit",
    data = .) %>%
~~~

````
    tabb()
```

```{r}
wildc %>%
  mutate(q = scale(q)) %>%
  filter(test == "m_turicensis") %>%
  glm(result ~ q + I(q^2) + age,
    family = binomial(), method = "brglmFit",
    data = .) %>%
    tabb()
```

```{r}
wildc %>%
  mutate(q = scale(q)) %>%
  filter(test == "m_turicensis") %>%
  glm(result ~ q + I(q^2) + mol_sex,
    family = binomial(), method = "brglmFit",
    data = .) %>%
    tabb()
```

```{r}
wildc %>%
  mutate(q = scale(q)) %>%
  filter(test == "m_turicensis") %>%
  glm(result ~ q + I(q^2) + factor(bcs>3),
    family = binomial(), method = "brglmFit",
    data = .) %>%
    tabb()
```
````
````

R script with pathogen richness added to relevant results obtained from previous code

```{r}
library(tidyverse) # for data manipulation and plotting
library(lme4) # for models
library(here) # makes file location easier
library(janitor) # for cleaning functions
library(broom) # to tidy model outpu
library(broom.mixed) # to tidy random effect models
library(patchwork) # combines plots
library(brglm2)
library(parameters)
library(gt)

conflicts_prefer(dplyr::filter)
```

Functions

Binomial test that fails gracefully if no data.

```{r}
binom.test2 <- possibly(binom.test, otherwise = list(conf.int = c(NA, NA)))

```

Import data

```{r}
wildc_wide <- read_csv(here("data", "wildc.csv"))

wildc <- wildc_wide %>%
  janitor::clean_names() %>%
```

```
    pivot_longer(c_m_haemominutum:b_bronchiseptica,
                 names_to = "test",
                 values_to = "result") %>%
  # drop snap tests

  filter(!str_detect(test, "snap")) |>

  # set SB as reference swa_pa
  mutate(swa_pa = fct_relevel(swa_pa, "SB")) %>%
  # drop NA results
  drop_na(result) |>
    # add n positive apart from the test in question

  group_by(swa_id) |>
  mutate(n_pos_total = sum(result)) |>
  ungroup() |>

  group_by(test, swa_pa) %>%

  # subtract test of interest from n_pos
  mutate(n_pos = n_pos_total - result) |>

  ungroup()


```

Plot regional prevalences

```
```{r, fig.height=10, fig.width=10}
  wildc %>%
  group_by(test, swa_pa) %>%
```
```

```
    summarise(r = sum(result, na.rm = TRUE),
              n = sum(!is.na(result)),
              prev = mean(r/n),
              lci = binom.test(r, n)$conf.int[[1]],
              uci = binom.test(r, n)$conf.int[[2]]) %>%
    ungroup() %>%
    ggplot() +
    aes(x = swa_pa, y = prev, ymin = lci, ymax = uci) +
    geom_pointrange(shape = 1) +
    facet_wrap(facets = ~ test, scale = "free", ncol = 3) +
    labs(title = "Prevalence by swa_pa/test",
         caption = "with 95% CI",
         x = "Swarm")
```

Models

Fit models

```{r}

# wildc <- wildc %>%
#   mutate(q = scale(q))

mod <- wildc %>%

  # mutate(q = scale(q)) %>%
  drop_na(result, q) %>%
  group_nest(test) %>%
  mutate(fit_1_m = map(data, ~ glmer(result ~ n_pos + q + (1 | swa_pa),
    family = binomial(), control=glmerControl(optimizer="bobyqa",
optCtrl=list(maxfun=100000)),
    data = .x,
```

```
  ))) %>%
  mutate(fit_2_m = map(data, ~ glmer(result ~ n_pos + q + I(q^2) + (1 | swa_pa),
    family = binomial(), control=glmerControl(optimizer="bobyqa",
optCtrl=list(maxfun=100000)),
    data = .x,
  ))) %>%
  mutate(fit_1_f = map(data, ~ glm(result ~ n_pos + q,
    family = binomial(), method = "brglmFit",
    data = .x
  ))) %>%
  mutate(fit_2_f = map(data, ~ glm(result ~ n_pos + q + I(q^2),
    family = binomial(), method = "brglmFit",
    data = .x
  )))


mod1 <- mod %>%
  pivot_longer(contains("fit"), names_to = "model", values_to = "fit") %>%

  mutate(aic = map_dbl(fit, AIC)) %>%
  mutate(singular = map_lgl(fit, performance::check_singularity)) %>%
  filter(!singular) %>%
  group_by(test) %>%
  mutate(delta_aic = aic - min(aic)) %>%
  filter(aic <= min(aic) + 2) %>%
  ungroup() %>%

    # manually choose simplest models within 2 AIC of minimum from fitted models above

  filter(test == "b_bronchiseptica" & model == "fit_1_f" |
         test == "c_m_haemominutum" & model == "fit_1_m" |
         test == "chlamydophila_felis" & model == "fit_1_f" |
         test == "fcv" & model == "fit_1_f" |
```

```
        test == "fe_lv" & model == "fit_1_f" |
        test == "fe_lv_snap" & model == "fit_1_f" |
        test == "fhv" & model == "fit_1_f" |
        test == "fiv" & model == "fit_1_f" |
        test == "fiv_snap" & model == "fit_1_f" |
        test == "m_haemofelis" & model == "fit_1_m" |
        test == "m_turicensis" & model == "fit_2_m" |
        test == "mycoplasma_felis" & model == "fit_1_f" |
        test == "tritrichomonas_foetus" & model == "fit_1_f")
```

Predict probability of positive vs q (using SB swa_pa as reference)

```{r}
mod1 %>%
  mutate(pred = map2(mod1$fit, mod1$data, ~predict(.x, newdata = .y %>% mutate(swa_pa = "SB", n_pos = 0)))) %>%
  filter(!str_detect(test, "snap")) |>
  unnest(c(data, pred)) %>%
  ggplot() +
  aes(x = q, y = plogis(pred)) +
  geom_line() +
  facet_wrap(~ test) +
  labs(x = "Q",
     y = "predicted prob(positive",
     title = "Predicted positivity vs Q",
     subtitle = "SB as reference swa_pa")

ggsave("predicted_prob_plot.pdf", height = 8, width = 8)

```

````
```{r}
mod1 %>%
  mutate(pred = map2(mod1$fit, mod1$data, ~predict(.x, newdata = .y %>% mutate(swa_pa = "SB")))) %>%
  unnest(c(data, pred)) %>%
  filter(test == "m_turicensis") %>%
  ggplot() +
  aes(x = q, y = plogis(pred)) +
  geom_line() +
  facet_wrap(~ test) +
  labs(x = "Q",
       y = "predicted prob(positive",
       title = "Predicted positivity vs Q",
       subtitle = "SB as reference swa_pa")
```
````

````
```{r}
# formatting criteria fro sci format
num_low <- 0.001
num_high <- 1000

mod1 %>%
  mutate(model_type = fct_recode(model,
                                 "mixed - linear" = "fit_1_m",
                                 "mixed - quadratic" = "fit_2_m",
                                 "fixed - linear" = "fit_1_f",
                                 "fixed - quadratic" = "fit_2_f")) %>%
  mutate(est = map(fit, parameters, exponentiate = TRUE)) %>%
  unnest(est) %>%
  mutate(Term = str_remove_all(Parameter, "I|\\(|\\)")) %>%
  discard(is.list) %>%
  filter(!str_detect(Parameter, "Intercept|SD")) %>%
````
````

select(Disease = test, `Model type` = model_type, Term, OR = Coefficient, CI_low, CI_high, `P-value` = p) %>%

mutate(Disease = fct_relevel(Disease, c("fiv", "fiv_snap", "fe_lv", "fe_lv_snap",

"fcv", "fhv", "chlamydophila_felis",

"b_bronchiseptica", "mycoplasma_felis", "m_haemofelis",

"c_m_haemominutum",

"m_turicensis", "tritrichomonas_foetus"))) %>%

arrange(Disease) %>%

gt::gt() %>%

gt::fmt_scientific(OR, rows = OR > num_high | OR < num_low) %>%

gt::fmt_number(OR, decimals = 3, rows = OR <= num_high & OR >= num_low) %>%

gt::fmt_scientific(CI_low, rows = CI_low > num_high | CI_low < num_low) %>%

gt::fmt_number(CI_low, decimals = 3, rows = CI_low <= num_high & CI_low >= num_low) %>%

gt::fmt_scientific(CI_high, rows = CI_high > num_high | CI_high < num_low) %>%

gt::fmt_number(CI_high, decimals = 3, rows = CI_high <= num_high & CI_high >= num_low) %>%

gt::fmt_number(`P-value`, decimals = 3) %>%

gt::cols_merge(OR:CI_high, pattern = "{1}, ({2} - {3})")

```

### Individual models

```{r}

tabb <- function(x){

x %>%

parameters::parameters(exponentiate = TRUE) %>%

mutate(Term = str_remove_all(Parameter, "I|\\(|\\)")) %>%

select(Term, OR = Coefficient, CI_low, CI_high, `P-value` = p) %>%

gt::gt() %>%

gt::fmt_scientific(OR, rows = OR > num_high | OR < num_low) %>%

gt::fmt_number(OR, decimals = 3, rows = OR <= num_high & OR >= num_low) %>%

```
  gt::fmt_scientific(CI_low, rows = CI_low > num_high | CI_low < num_low) %>%
  gt::fmt_number(CI_low, decimals = 3, rows = CI_low <= num_high & CI_low >= num_low)
%>%
  gt::fmt_scientific(CI_high, rows = CI_high > num_high | CI_high < num_low) %>%
  gt::fmt_number(CI_high, decimals = 3, rows = CI_high <= num_high & CI_high >=
num_low) %>%
  gt::fmt_number(`P-value`, decimals = 3) %>%
  gt::cols_merge(OR:CI_high, pattern = "{1}, ({2} - {3})")
}
```

FCV

```{r}
wildc %>%
  filter(test == "fcv") %>%
  glm(result ~ q + mol_sex,
    family = binomial(), method = "brglmFit",
    data = .) %>%
  tabb()
```

```{r}
wildc %>%
  filter(test == "fcv") %>%
  glm(result ~ q + factor(bcs>3),
    family = binomial(), method = "brglmFit",
    data = .) %>%
tabb()
```

```{r}
wildc %>%
```

````
  filter(test == "fcv") %>%
  glm(result ~ q + age,
    family = binomial(), method = "brglmFit",
    data = .) %>%
  tabb()
```

```{r}
wildc %>%
  filter(test == "fcv") %>%
  glm(result ~ q + social_sys,
    family = binomial(), method = "brglmFit",
    data = .) %>%
  tabb()
```

### FIV

```{r}
wildc %>%
  filter(test == "fiv") %>%
  glm(result ~ q + social_sys,
    family = binomial(), method = "brglmFit",
    data = .) %>%
  tabb()
```

```{r}
wildc %>%
  filter(test == "fiv") %>%
  glm(result ~ q + mol_sex,
    family = binomial(), method = "brglmFit",
````

```
    data = .) %>%
  tabb()
```

```{r}
wildc %>%
  filter(test == "fiv") %>%
  glm(result ~ q + factor(bcs>3),
    family = binomial(), method = "brglmFit",
    data = .) %>%
tabb()
```

```{r}
wildc %>%
  filter(test == "fiv") %>%
  glm(result ~ q + age,
    family = binomial(), method = "brglmFit",
    data = .) %>%
tabb()
```

m_turicensis

```{r}
wildc %>%
  filter(test == "m_turicensis") %>%
  glmer(result ~ q + I(q^2) + (1 | swa_pa) + mol_sex,
    family = binomial(), control=glmerControl(optimizer="bobyqa",
optCtrl=list(maxfun=100000)),
    data = .) %>%
    tabb()
```

````
```

```{r}
wildc %>%
  filter(test == "m_turicensis") %>%
  glmer(result ~ q + I(q^2) + (1 | swa_pa) + factor(bcs>3),
    family = binomial(), control=glmerControl(optimizer="bobyqa",
optCtrl=list(maxfun=100000)),
    data = .) %>%
    tabb()

```

```{r}
wildc %>%
  filter(test == "m_turicensis") %>%
  glmer(result ~ q + I(q^2) + (1 | swa_pa) + age,
    family = binomial(), control=glmerControl(optimizer="bobyqa",
optCtrl=list(maxfun=100000)),
    data = .) %>%
    parameters::parameters(exponentiate = TRUE)

```

```{r}
wildc %>%
  filter(test == "m_turicensis") %>%
  glmer(result ~ q + I(q^2) + (1 | swa_pa) + social_sys,
    family = binomial(), control=glmerControl(optimizer="bobyqa",
optCtrl=list(maxfun=100000)),
    data = .) %>%
    parameters::parameters(exponentiate = TRUE)
````

```

### mycoplasma_felis

```{r}
wildc %>%
  filter(test == "mycoplasma_felis") %>%
  glm(result ~ q + mol_sex,
    family = binomial(), method = "brglmFit",
    data = .) %>%
  parameters::parameters(exponentiate = TRUE)
```

```{r}
wildc %>%
  filter(test == "mycoplasma_felis") %>%
  glm(result ~ q + factor(bcs>3),
    family = binomial(), method = "brglmFit",
    data = .) %>%
  parameters::parameters(exponentiate = TRUE)
```

```{r}
wildc %>%
  filter(test == "mycoplasma_felis") %>%
  glm(result ~ q + age,
    family = binomial(), method = "brglmFit",
    data = .) %>%
  parameters::parameters(exponentiate = TRUE)
```

```{r}

~~~~
wildc %>%
  filter(test == "mycoplasma_felis") %>%
  glm(result ~ q + social_sys,
    family = binomial(), method = "brglmFit",
    data = .) %>%
  parameters::parameters(exponentiate = TRUE)
```

m_haemofelis

```{r}
wildc %>%
  filter(test == "m_haemofelis") %>%
  glm(result ~ q + mol_sex,
    family = binomial(), method = "brglmFit",
    data = .) %>%
  parameters::parameters(exponentiate = TRUE)
```

```{r}
wildc %>%
  filter(test == "m_haemofelis") %>%
  glm(result ~ q + factor(bcs>3),
    family = binomial(), method = "brglmFit",
    data = .) %>%
  parameters::parameters(exponentiate = TRUE)
```

```{r}
wildc %>%
  filter(test == "m_haemofelis") %>%
  glm(result ~ q + age,
~~~~

~~~
    family = binomial(), method = "brglmFit",
    data = .) %>%
  parameters::parameters(exponentiate = TRUE)
```

```{r}
wildc %>%
  filter(test == "m_haemofelis") %>%
  glm(result ~ q + social_sys,
    family = binomial(), method = "brglmFit",
    data = .) %>%
  parameters::parameters(exponentiate = TRUE)
```

Other models

```{r}
wildc %>%
  filter(test == "c_m_haemominutum") %>%
  glm(result ~ q + age,
    family = binomial(), method = "brglmFit",
    data = .) %>%
  parameters::parameters(exponentiate = TRUE)
```

Check c_m_haemominutum model

```{r}
lmtest::lrtest(
  wildc %>%
    drop_na(q) %>%
  filter(test == "c_m_haemominutum") %>%
~~~

```
  glm(result ~ 1 + swa_pa,
      family = binomial(),
    data = .),
  wildc %>%
  drop_na(q) %>%
  filter(test == "c_m_haemominutum") %>%
  glm(result ~ q + I(q^2) + swa_pa,
      family = binomial(),
    data = .)
)

```

```{r}
wildc %>%
  filter(test == "fcv") %>%
  glm(result  ~ q,
    family = binomial(),
    data = .) %>%
  parameters::parameters(exponentiate = TRUE)
```

```{r}
wildc %>%
  filter(test == "fcv") %>%
  # filter(social_sys == "Solitary") %>%
  glm(result  ~ q,
    family = binomial(),
    data = .) %>%
  parameters::parameters(exponentiate = TRUE)
```

Social system models

```{r}
wildc %>%

  # we need to scale q to have model run

  drop_na(result, q) %>%
  group_nest(test) %>%
  mutate(fit_1_m = map(data, ~ glmer(result ~ social_sys + (1 | swa_pa),
    family = binomial(), control=glmerControl(optimizer="bobyqa",
optCtrl=list(maxfun=100000)),
    data = .x,
  ))) %>%
  mutate(fit_1_f = map(data, ~ glm(result ~ social_sys,
    family = binomial(), method = "brglmFit",
    data = .x
  ))) %>%

  pivot_longer(contains("fit"), names_to = "model", values_to = "fit") %>%

  mutate(aic = map_dbl(fit, AIC)) %>%
  mutate(singular = map_lgl(fit, performance::check_singularity)) %>%
  filter(!singular) %>%
  group_by(test) %>%
  mutate(delta_aic = aic - min(aic)) %>%
  filter(aic <= min(aic) + 2) %>%
  ungroup() %>%

    filter(test == "b_bronchiseptica" & model == "fit_1_f" |
           test == "c_m_haemominutum" & model == "fit_1_m" |
           test == "chlamydophila_felis" & model == "fit_1_f" |
           test == "fcv" & model == "fit_1_f" |
```

```
        test == "fe_lv" & model == "fit_1_f" |
        test == "fe_lv_snap" & model == "fit_1_f" |
        test == "fhv" & model == "fit_1_f" |
        test == "fiv" & model == "fit_1_f" |
        test == "fiv_snap" & model == "fit_1_f" |
        test == "m_haemofelis" & model == "fit_1_m" |
        test == "m_turicensis" & model == "fit_1_m" |
        test == "mycoplasma_felis" & model == "fit_1_f" |
        test == "tritrichomonas_foetus" & model == "fit_1_f") %>%

  mutate(model_type = fct_recode(model,
                     "mixed - linear" = "fit_1_m",
                     "mixed - quadratic" = "fit_2_m",
                     "fixed - linear" = "fit_1_f",
                     "fixed - quadratic" = "fit_2_f")) %>%
  mutate(est = map(fit, parameters, exponentiate = TRUE)) %>%
  unnest(est) %>%
  mutate(Term = str_remove_all(Parameter, "I|\\(|\\)")) %>%
  discard(is.list) %>%
  filter(!str_detect(Parameter, "Intercept|SD")) %>%
  select(Disease = test, `Model type` = model_type, aic, Term, OR = Coefficient, CI_low,
CI_high, `P-value` = p) %>%
  mutate(Disease = fct_relevel(Disease, c("fiv", "fiv_snap", "fe_lv", "fe_lv_snap",
                          "fcv", "fhv", "chlamydophila_felis",
                          "b_bronchiseptica", "mycoplasma_felis", "m_haemofelis",
                          "c_m_haemominutum",
                          "m_turicensis", "tritrichomonas_foetus"))) %>%
  arrange(Disease) %>%
  gt::gt() %>%
  gt::fmt_scientific(OR, rows = OR > num_high | OR < num_low) %>%
  gt::fmt_number(OR, decimals = 3, rows = OR <= num_high & OR >= num_low) %>%
  gt::fmt_scientific(CI_low, rows = CI_low > num_high | CI_low < num_low) %>%
```

````
  gt::fmt_number(CI_low, decimals = 3, rows = CI_low <= num_high & CI_low >= num_low) %>%
  gt::fmt_scientific(CI_high, rows = CI_high > num_high | CI_high < num_low) %>%
  gt::fmt_number(CI_high, decimals = 3, rows = CI_high <= num_high & CI_high >= num_low) %>%
  gt::fmt_number(`P-value`, decimals = 3) %>%
  gt::cols_merge(OR:CI_high, pattern = "{1}, ({2} - {3})")
```


# Use scaled Q for the quadratic


```{r}
wildc %>%
  mutate(q = scale(q)) %>%
  filter(test == "m_turicensis") %>%
  glm(result ~ q + I(q^2) + n_pos,
    family = binomial(), method = "brglmFit",
    data = .) %>%
    tabb()
```


```{r}
wildc %>%
  mutate(q = scale(q)) %>%
  filter(test == "m_turicensis") %>%
  glm(result ~ q + I(q^2) + social_sys,
    family = binomial(), method = "brglmFit",
    data = .) %>%
    tabb()
```


```{r}
wildc %>%
````

```
  mutate(q = scale(q)) %>%
  filter(test == "m_turicensis") %>%
  glm(result ~ q + I(q^2) + age,
    family = binomial(), method = "brglmFit",
    data = .) %>%
    tabb()
```

```{r}
wildc %>%
  mutate(q = scale(q)) %>%
  filter(test == "m_turicensis") %>%
  glm(result ~ q + I(q^2) + mol_sex,
    family = binomial(), method = "brglmFit",
    data = .) %>%
    tabb()
```

```{r}
wildc %>%
  mutate(q = scale(q)) %>%
  filter(test == "m_turicensis") %>%
  glm(result ~ q + I(q^2) + factor(bcs>3),
    family = binomial(), method = "brglmFit",
    data = .) %>%
    tabb()
```

Plot q vs npostotal

```{r}
wildc |>
```

```
  select(swa_id, q, n_pos_total) |>
  distinct() |>
  ggplot() +
  aes(x = q, y = n_pos_total) +
  geom_smooth(se = TRUE, colour = "grey60", span = 0.8) +
  geom_point(position = position_jitter(height = 0.05, width = 0),
             shape = 1) +
  labs(x = "Q", y = "Total number of positive tests") +
  theme_bw(base_size = 14)

ggsave("n_pos_total_vs_q.eps", height = 6, width = 8)
```
```
```
